# Honey Bees as Bioindicators of Environmental PFASs Contamination and Potential Effects on Gut-Microbial Communities

**DOI:** 10.3390/insects17101027

**Published:** 2026-10-06

**Authors:** Laura Zulian, Arianna Peruzzo, Michela Bertola, Gioele Scopel, Elena Albertin, Marianna Martinello, Chiara Manzinello, Alessandra Moressa, Sara Petrin, Federica Gallocchio, Mauro Dacasto, Marianna Pauletto, Franco Mutinelli, Fulvio Bordin, Anna Granato

**Affiliations:** 1 National Reference Laboratory for Honey Bee Health, Istituto Zooprofilattico Sperimentale delle Venezie, 35020 Legnaro, Italy; lzulian@izsvenezie.it (L.Z.); mbertola@izsvenezie.it (M.B.); ealbertin@izsvenezie.it (E.A.); mmartinello@izsvenezie.it (M.M.); cmanzinello@izsvenezie.it (C.M.); fmutinelli@izsvenezie.it (F.M.); 2 Laboratory of Microbial Ecology and Genomics, Istituto Zooprofilattico Sperimentale delle Venezie, 35020 Legnaro, Italy; aperuzzo@izsvenezie.it (A.P.); spetrin@izsvenezie.it (S.P.); 3 Department of Chemistry, Istituto Zooprofilattico Sperimentale delle Venezie, 35020 Legnaro, Italy; gscopel@izsvenezie.it (G.S.); amoressa@izsvenezie.it (A.M.); fgallocchio@izsvenezie.it (F.G.); 4 Department of Comparative Biomedicine and Food Science, University of Padova, 35020 Legnaro, Italy; mauro.dacasto@unipd.it (M.D.); marianna.pauletto@unipd.it (M.P.)

**Keywords:** honey bees, PFAS, bioindicator, gut-microbial communities, pesticides, metagenomics, environmental pollutants

## Abstract

Honey bees are valuable bioindicators of environmental contamination due to their wide foraging range and exposure to diverse biotic and abiotic stressors. This study evaluates the use of honey bees as bioindicators for detecting per- and polyfluoroalkyl substances (PFASs) in areas with varying levels of exposure risk (high-, intermediate-, and low-), as well as their potential effects on gut-microbial communities. Analytical chemistry to detect PFASs and pesticide residues were combined with molecular and metagenomic analyses to identify common honey bee pathogens and characterize gut-microbial communities. Short-chain PFASs were predominated over long-chain compounds, particularly in high-risk areas, while pesticide residues occurred in only a few apiaries. Molecular analyses revealed several widespread pathogens, while metagenomics showed a stable gut microbiome mainly composed of the genera *Lactobacillus*, *Bartonella*, *Bombilactobacillus*, *Snodgrassella*, *Gilliamella*, *Bifidobacterium*, and *Gluconacetobacter*. However, microbial-community composition differed significantly between apiaries with high- versus low-risk PFAS exposure. These results support honey bees as effective bioindicators for environmental PFAS monitoring and suggest that PFAS exposure may be associated with alterations in their gut-microbial communities. Further research is needed to elucidate the impact of PFASs on honey bee toxicity, gut-microbiota composition, and overall health.

## Simple Summary

Honey bees (*Apis mellifera*) are vital pollinators, playing a key role in maintaining global ecosystems and biodiversity; furthermore, their high sensitivity to environmental changes makes them valuable bioindicators. The widespread presence of environmental contaminants, such as per- and polyfluoroalkyl substances, is a growing concern for ecosystem health due to their high environmental persistence and potential toxic effects on living organisms. In this study, honey bees were used as bioindicators to detect these sub-stances in areas with different exposure-risk levels. Analyses revealed these compounds in bee samples, showing clear differences in concentration and chemical profile among sampling sites, thereby reflecting varying levels of environmental contamination. Additionally, we evaluated whether exposure to these compounds could alter the honey bees’ gut-bacterial communities, which are strictly involved in maintaining insect health. Significant shifts in gut-microbial community composition were detected in apiaries exposed to a higher risk of exposure, suggesting a potential negative impact of these contaminants. Overall, these findings support the use of honey bees as bioindicators for monitoring emerging contaminants and for assessing chemical pressure on ecosystems.

## 1. Introduction

Honey bees (*Apis mellifera*) are vital pollinators, playing a key role in maintaining global ecosystems and biodiversity. Despite this, in recent decades a decline in honey bee populations has been recorded worldwide. These losses result from the continuous and direct interaction of *A. mellifera* with nature across diverse habitats, and arise from multiple abiotic and biotic stressors. Abiotic factors include climate change, which can alter plant flowering periods affecting the availability of nutritional resources; intensive agriculture and monocultures; and the widespread use of pesticides as well as the exposure to environmental pollutants or contaminants. With respect to biotic pressures, a number of honey bee parasites and pathogens, such as *Varroa destructor* and deformed wing virus (DWV), can critically impair honey bee health [1,2,3].

Environmental pollution is a widely recognized abiotic factor severely threatening honey bees and other pollinators, compromising their ecological services. The use of chemicals in crop production has long been considered essential, and evidence supporting the adverse impact of pesticides on honey bee health is increasing, particularly for neonicotinoid insecticides [4,5]. In addition, sub-lethal effects of anthropogenic compounds (even in different combinations) on bees have been assessed both in the field (in situ, in vivo) and under laboratory (in vitro) conditions, using data from individual beekeepers or well-established Organization for Economic Co-operation and Development (OECD)*-*approved regulatory test systems for evaluating acute and chronic toxicity. Honey bees are therefore currently considered effective bioindicators of environmental health and potentially helpful for environmental risk assessments [6,7,8,9,10]. Numerous studies highlight the effective application of bees in environmental monitoring programs, demonstrating their capacity to serve as reliable bioindicators of pollution from pesticides [8,11,12], heavy metals [11,13,14,15], and microplastics [10,16].

While the impact of pesticides on honey bee health has been extensively studied, much less is known about the effects of the so-called “emerging contaminants”. Among the latter, per- and polyfluoroalkyl substances (PFASs) have recently raised considerable concern due to their environmental persistence, widespread distribution, and potential toxicity [17]. PFASs are synthetic molecules first produced in the 1940s. They feature a hydrophobic and lipophilic carbon chain, where fluorine atoms replace hydrogen atoms on some or all of the carbons. Based on their functional groups, they can be grouped into various classes and subclasses, with a primary distinction between “long-chain” or “legacy” PFASs and “short-chain” or “alternative” PFASs [17,18,19]. The term “alternative” was introduced under the assumption that these compounds were less toxic and persistent than legacy PFASs. Nevertheless, growing evidence has demonstrated that they share similar persistence and toxicological profiles, a phenomenon referred to as “regrettable substitution” [20]. Due to their high chemical and thermal stability deriving from strong C–F bonds, PFASs have been widely used in textile coatings, surfactants, pesticides, food-contact packaging, firefighting foams, and cosmetics [21,22,23,24,25]. Their exceptional chemical and thermal stability is responsible for their extreme persistence in the environment (soil, water, and atmosphere), earning them the designation of “forever chemicals” [24,25,26]. As a consequence of their persistence, bioaccumulation in animal and human tissues is also observed [27], raising growing concerns about their impacts on both ecosystem and human health [28].

In Italy, the PFAS environmental-monitoring plan is coordinated nationally by the National System for Environmental Protection (SNPA), and implemented locally by the Regional Environmental Protection Agencies (ARPAs). Based on PFAS occurrence in environmental and water matrices and estimated exposure levels, the Veneto Region has been classified into four exposure zones: (a) Red zone (highest PFAS concentrations and the greatest health concern); (b) Orange zone (caution for potentially contaminated private wells and water intakes); (c) Yellow zone (enhanced water monitoring); (d) Green zone (PFASs detected not at critical levels only in selected environmental matrices; zone used to study spatial dispersion and temporal evolution of PFAS contamination) [29]. These exposure zones provide a valuable framework for biomonitoring studies using biological matrices, including pollinators and particularly honey bees, across a gradient of environmental PFAS contamination.

The convenience of honey bees in PFAS environmental risk assessment lies in multiple lines of evidence. First, bees can be reared across a wide range of geographical areas, making their exposure patterns and contaminant bioaccumulation strongly dependent on local environmental conditions. Second, worker bees usually forage within a 2–3 km radius from the hive [30,31], making them effective bioindicators of local environmental contamination [32,33,34,35]. After foraging, bees transport nectar, pollen, water and any collected pollutants back to the hive, where these substances accumulate in hive products such as wax and honey [33,36,37]. Honey bees and hive products can therefore provide valuable information on the occurrence and distribution of environmental pollutants, including PFASs [5,8].

In environmental toxicology, exposure routes characterize how xenobiotics enter living organisms, shaping health risks, and guiding risk assessment. As in mammals [38], bees can be exposed to environmental contaminants through (a) oral ingestion of contaminated water, nectar, and pollen; (b) direct contact through the exoskeleton; and (c) aerial exposure to airborne particulates or chemicals [39]. In terms of toxicokinetic relevance, the gut represents the primary organ responsible for absorption and the first site of interaction with xenobiotics [40].

Human exposure to PFASs occurs primarily via oral routes, through involuntary ingestion of contaminated drinking water, food, and household dust [41]. Within the gastrointestinal tract, PFASs (and other environmental pollutants, e.g., pesticides, heavy metals, nitrosamines) interact with the gut environment, which shapes their bioavailability and toxicity [41,42]. The gut microbiota is known to be a crucial regulator of human health, playing a fundamental role in metabolism, immune function, and oxidative homeostasis [43] and it critically influences contaminant toxicity by interacting with xenobiotics before or after absorption [42,44]. Indeed, many xenobiotics have been shown to significantly affect the composition of the gut bacterial communities, often leading to dysbiosis and oxidative stress. PFAS exposure has been associated with alterations in gut physiology, including epithelial barrier dysfunction, immune imbalance, and changes in microbiota composition [41]. Crucially, PFAS-induced microbial-community remodeling is mediated not exclusively by its direct toxic effect, but also by a cascade of indirect, systemic host mechanisms involved in chronic diseases [45]. Although the gut microbiota’s metabolic capacity is well established in relation to endogenous compounds, its role in the first-pass metabolism of environmental pollutants is an emerging area of research [42,46]. Thus, the gut microbiota plays a crucial role in how environmental chemicals affect health and studies investigating the impact of PFASs on gut microbiota are emerging [45].

As in mammals, the honey bee’s gut microbiota plays an essential role in health, influencing development, behavior, nutrition, immune function, and detoxification [47]; furthermore, it protects against pathogens, including opportunistic bacteria, fungi, parasites, and viruses [48,49], enhancing host immunity by upregulating genes involved in antimicrobial peptide production and in the melanization pathway. This innate system protects against pathogens via humoral responses and cellular mechanisms like phagocytosis, although the molecular mechanisms underlying microbiota-mediated immune modulation remain only partially understood [49]. Beyond immunity, the gut microbiota contributes to host development by influencing weight gain, endocrine regulation, cognitive performance, and modulating the gut–brain axis, ultimately affecting social behavior within the colony. It also plays a central role in metabolism, participating in fatty acid and amino acid biosynthesis, carbohydrate degradation, and the breakdown of complex plant polysaccharides and secondary metabolites derived from nectar and pollen [49]. Perturbation of the honey bee gut microbiome has been consistently demonstrated to impair bee health and reduce colony fitness. Biotic stressors, such as the microsporidium *Vairimorpha ceranae* (recently phylogenetically reclassified from the genus *Nosema* to *Vairimorpha*) [50,51,52] and DWV, have been shown to alter the bacterial community composition [53,54,55,56,57]. In addition, DWV can induce host immunosuppression accompanied by gut dysbiosis, resulting in shifts in the relative abundance of both core and non-core bacterial taxa and a decline in beneficial bacterial genera [58].

Exposure to environmental contaminants also represent an important driver of gut microbiota dysbiosis. Pesticide exposure can alter the composition and diversity of the bee gut microbiota, causing microbial-community imbalance and reducing beneficial microorganisms, either directly by impairing bacterial growth or indirectly by affecting gut microbiota homeostasis [59,60,61]. These fluctuations are further influenced by pesticide formulation, concentration, and exposure duration [60,62,63]. Notably, the effects of PFAS exposure on honey bee health and gut microbiota remain largely unexplored. Few available studies indicate that PFASs adversely affect honey bee colonies with impacts on brood production, worker viability, behavior, and temperament. Moreover, PFASs accumulate in honey bee tissues and may be introduced into the hive through contaminated nectar and pollen collected during foraging [4,37,64,65,66]. Therefore, the objectives of the present study were: (a) to use honey bees as bioindicators to assess environmental PFAS contamination in apiaries located in different areas of risk exposure from the Veneto Region (Italy); and (b) to investigate the impact of PFAS exposure on honey bee gut-microbiota composition. To this end, honey bee samples will be analyzed to evaluate the presence of PFASs using validated analytical chemistry methods, while gut-microbial communities will be characterized by metagenomics. Furthermore, PFAS analytical and microbiome structural data will be integrated with common honey bee pesticides and pathogens to investigate potential associations. Through this approach we aimed to support the use of honey bees as sentinels for environmental PFAS contamination and to contribute to a better understanding of the potential effects of PFASs on pollinator health, with implications for environmental monitoring and risk assessment in contaminated regions.

## 2. Materials and Methods

### 2.1. Apiary Selection and Sampling

Based on the availability of local beekeepers, 15 apiaries, each comprising at least 10 hives, were selected in the Veneto Region (north-eastern Italy). The apiaries were located either within the red and yellow zones identified in Annex 1 to Veneto Regional Government Resolution No. 691 of 21 May 2018 [29], or outside the areas officially designated as affected by PFAS contamination. The latter apiaries were included as a low-contamination reference group. More detailed information on PFAS concentrations in surface waters near each apiary was obtained from the online map provided by the Regional Agency for Environmental Prevention and Protection of Veneto (ARPAV; https://gaia.arpa.veneto.it/maps/1299— accessed on 20 August 2026) [67]. Based on the combined concentrations of PFOA, PFOS, perfluorononanoic acid (PFNA), and perfluorohexane sulfonic acid (PFHxS), the apiaries were assigned to three exposure groups: high (red area; 56–740 ng L^−1^), intermediate (yellow area; 5–50 ng L^−1^) and low (areas outside the officially designated red and yellow zones and identified as brown; 0.9–6 ng L^−1^). Seven apiaries belonged to the high-exposure group (H) and were all located in the Province of Vicenza (H-VI-1 to H-VI-7). Four apiaries belonged to the intermediate-exposure group (I): three were located in the Province of Padua (I-PD-1 to I-PD-3) and one in the Province of Vicenza (I-VI-8). The remaining four apiaries constituted the low-exposure reference group (L) and were located in the provinces of Padua (L-PD-4), Vicenza (L-VI-9 and L-VI-10), and Verona (L-VR-1) (Figure 1).

The selected apiaries were located within a maximum of 5 km from the ARPAV monitoring site. *A. mellifera* typically exhibits a foraging radius of 2 to 3 km around their hive; however, individuals can extend their flight distance up to 8 km under conditions of scarce floral resources.

Prior to sampling, a consent form and a questionnaire were completed in cooperation with the beekeepers to collect specific apiary data, including information on the apiary itself (e.g., geo-referencing, territory type, agronomic and vegetation characteristics, health status, etc.), colony strength (assessed via on-site inspections during sampling and anamnestic data collection), and the beekeeper’s management practices (e.g., control measures against *Varroa* mite infestation and other treatments).

Sampling was carried out in July 2024, during the period of maximum seasonal activity. Forager honey bees were collected from the flight boards of one central hive of each apiary, excluding “head hives”, since they receive a greater number of returning foragers from other hives due to drift phenomenon. Approximately 300 honey bees per apiary were sampled. They were immediately frozen on-site using dry ice and subsequently stored at −80 °C until analysis.

### 2.2. PFAS Residues Analysis

Target analysis encompasses 14 per- and polyfluoroalkyl substances (PFASs), categorized into short-chain (PFBA, PFPeA, PFHxA, PFHpA, PFBS, PFHxS, GenX, and C_6_O_4_) and long-chain compounds (PFOA, PFOS, PFNA, PFDA, PFUnA, and PFDoA). Quantification was achieved via liquid chromatography–tandem mass spectrometry (LC–MS/MS) utilizing isotope dilution with corresponding ^13^C-labeled internal standards. Whole honey bees were analyzed using a modified QuEChERS extraction with C18/PSA dispersive solid-phase extraction (dSPE) cleanup, adapted from previous residue analysis protocols [68,69].

Briefly, honey bees collected from each of the 15 apiaries were homogenized with an Ultra-Turrax^®^ T25 Digital homogenizer (IKA^®^-Werke GmbH & Co. KG, Staufen, Germany) and 1.0 g of homogenate was used for extraction. The sample was fortified with 5 μL of mass-labeled PFASs mixture (10 ng mL^−1^), extracted with acetonitrile using Quick, Easy, Cheap, Effective, Rugged, and Safe (QuEChERS) salts, and cleaned up by dispersive SPE (PSA/C18/MgSO_4_) using tubes containing 25 mg PSA, 25 mg C18, and 150 mg MgSO_4_. Extracts were evaporated under a gentle stream of N_2_ and reconstituted in 120 μL of 10 mM ammonium acetate in 80% MeOH/20% ACN prior to LC MS/MS analysis. PFAS analysis was performed in a single replicate.

The Analyst software AB SCIEX V 1.6.3 (Framingham, MA, USA) was used to control the LC–MS system and the MultiQuant 3.0.2 software (Framingham, MA, USA) was used to quantify analytes.

LC–MSMS method details, method validation, and quality control (e.g., recovery and repeatability measurements) [70] are provided in the Supplementary Materials (Tables S1 and S2).

### 2.3. Pesticide Residues Analysis

Pesticide residues in honey bees collected from each of the 15 apiaries were evaluated according to Martinello et al. (2021) [12]. Briefly, honey bees were ground into a fine powder using a crushing mill (A11 basic, IKA-Werke GmbH & Co. KG, Staufen, Germany) cooled with liquid nitrogen. The homogenized sample (2 g) was extracted in duplicate and purified using the QuEChERS EN method and dispersive SPE Fatty Samples EN salts. Extracts were subsequently analyzed by both liquid and gas chromatography coupled with triple-quadrupole tandem mass spectrometry (LC–MS/MS and GC–MS/MS, respectively) to screen 292 pesticide residues (listed in Supplementary Table S3). Pesticide analysis was performed in a single replicate and the results were expressed in µg/bee.

### 2.4. Honey Bee Pathogens Investigation

Eleven common honey bee pathogens, including fungi, protozoa, and viruses, were investigated using biomolecular methods.

To detect the fungi *Vairimorpha apis*, *V. ceranae*, as well as the trypanosomatids *Crithidia mellificae* and *Lotmaria passim*, a pool of 60 honey bees per apiary was homogenized in 60 mL of sterile phosphate-buffered saline (PBS). The presence of spores of *Vairimorpha* spp. was initially evaluated by examining approximately 20 μL of homogenate under a microscope at 400× magnification. Only the samples tested positive underwent polymerase chain reaction (PCR) analysis for species identification (*V. apis*/*V. ceranae*). DNA extraction was performed using the QIAamp DNA Mini kit (Qiagen, Hilden, Germany), according to the manufacturer’s instructions. Qualitative (A_260_/A_280_ and A_260_/A_230_ nm absorbance ratios) and quantitative (ng/μL) evaluations of the isolated DNA were performed using a Nanodrop™ One^C^ spectrophotometer (Thermo Fisher Scientific, Waltham, MA, USA). DNA samples were stored at −20 °C until analysis.

PCR assays (including primer sequences, concentrations, and amplification conditions) were carried out as described by Bordin et al. (2026) [71] and the resulting amplification products were then analyzed by capillary electrophoresis on a LabChip GX Touch HT^®^ instrument (Perkin Elmer, Waltham, MA, USA).

For virus detection, a pool of five honey bees per apiary was analyzed after homogenization using a Tissue Lyser II (Qiagen, Hilden, Germany) for 2 cycles of 1 min each, at 30 Hz in the presence of buffer RA1 and a 5 mm stainless steel bead. RNA extraction was performed using the NucleoSpin RNA Kit (Macherey-Nagel, Düren, Germany) according to the manufacturer’s instructions. Qualitative (A260/A280 and A260/A230 nm absorbance ratios) and quantitative (ng/μL) evaluations of extracted RNA were performed using a NanoDrop™ One^C^ spectrophotometer (Thermo Fisher Scientific, Waltham, MA, USA). RNA samples were stored at −80 °C until analysis.

Several honey bee RNA viruses were investigated following the biomolecular protocols (including primer and probe sequences, concentrations, and amplification conditions) previously described by Bordin et al. (2022) [72] except for DWV, for which the protocol published by Martinello et al. (2017) [73] was used. Specifically, the acute bee-paralysis virus (ABPV), black queen cell virus (BQCV), chronic bee-paralysis virus (CBPV), DWV, and sacbrood virus (SBV) were screened using one-step real-time RT-PCR (rRT-PCR). Conversely, the Kashmir bee virus (KBV) and Israeli acute-paralysis virus (IAPV) were investigated using one-step endpoint RT-PCR and the amplification products were analyzed by capillary electrophoresis on a LabChip GX Touch HT^®^ instrument.

To ensure the reliability of the molecular assays and monitor for potential contamination, negative process controls were included in each DNA and RNA extraction session, while both negative and positive controls were run in parallel during each PCR amplification.

### 2.5. 16S rRNA Gene Sequencing and Bioinformatics Analyses

All 15 sampled apiaries were subjected to analytical and biomolecular investigations to detect the presence of PFASs, pesticides, and common honey bee pathogens. To investigate the potential impact of PFAS exposure on the honey bee gut microbiota, a subset of apiaries was selected for metagenomic analysis based on the PFAS analytical results. Specifically, two apiaries were selected from each of the three PFAS exposure risk areas, resulting in a total of six apiaries included in the metagenomic analysis (Red, high-risk, H-VI-1, H-VI-7; Yellow, intermediate-risk, I-PD-2, I-PD-3; Brown, low-risk, L-VI-9, L-VI-10).

For each apiary, the gut of 10 adult honey bees was dissected under a stereomicroscope using sterile tweezers. Dissected guts from bees from the same apiary were pooled, resulting in one pooled sample per apiary. Each pooled gut sample was then divided into three aliquots, and DNA extraction was performed independently on each aliquot, resulting in three technical extraction replicates per apiary. Total DNA was extracted using the QIAamp Microbiome Host-Free Kit (Qiagen, Hilden, Germany), following the manufacturer’s instructions.

Extracted DNA was used as a template for the amplification of the V3–V4 hypervariable regions of the bacterial *16S rRNA* gene. Amplicon libraries were prepared according to the Illumina 16S Metagenomic Sequencing Library Preparation protocol, using the primers Bact341F and Bact785R (Fwd: CCTACGGGNGGCWGCAG; Rev: GACTACHVGGGTATCTAATCC) previously described by Klindworth et al. (2013) [74], using the Nextera XT DNA Library Prep kit (Illumina, San Diego, CA, USA). PCR was carried out under the following thermal conditions: initial denaturation at 95 °C for 3 min, followed by 30 cycles of denaturation at 95 °C for 30 s, annealing at 55 °C for 30 s, and extension at 72 °C for 30 s. A final elongation step was performed at 72 °C for 5 min. The successful amplification was verified by electrophoresis on a 2% Tris-Acetate-EDTA (TAE) agarose gel, confirming the presence of an amplicon of approximately 550 bp.

Library concentration and quality were assessed using the dsDNA High Sensitivity Kit for the Qubit™ 2.0 fluorimeter (Thermo Fisher Scientific, Waltham, MA, USA) and the Agilent TapeStation 2200 System (Agilent Technologies, Santa Clara, CA, USA), respectively. Libraries were equimolarly pooled and sequenced on an Illumina MiSeq™ instrument with Illumina MiSeq™ 600 V3 kit (600-cycle cartridge) (Illumina, San Diego, CA, USA), generating 2 × 300 bp paired-end reads.

Raw sequencing data were processed in RStudio (version R4.3.0—RStudio Team, 2020) using the DADA2 package [75]. Briefly, forward and reverse reads were filtered and trimmed according to their quality profiles. Filtered reads were then dereplicated to collapse identical sequencing reads into unique sequences. Amplicon sequence variants (ASVs) were inferred, and forward and reverse reads were merged to reconstruct the full-denoised sequences. Chimeric sequences were subsequently identified and removed to improve the accuracy of downstream analyses. Taxonomic assignment of the resulting ASVs was performed using the SILVA ribosomal RNA gene database (version 138.2) [76]. Because V3–V4 *16S rRNA* gene amplicon sequencing does not consistently provide sufficient discriminatory power for unambiguous species-level assignment, downstream taxonomic analyses, including PFAS–microbiota correlation analyses, were conservatively performed at the genus level to avoid over-classification [77]. Statistical analyses were performed using the phyloseq package in R [78]. The DNA raw data generated in this study have been deposited in the Sequence Read Archive (SRA) database (https://www.ncbi.nlm.nih.gov/sra—accessed on 14 August 2026) under the accession number PRJNA1512439.

Following the methodology described by Braglia et al. [79], the study-specific core microbiome was defined using prevalence and mean relative-abundance criteria. Technical extraction replicates were first averaged within each biologically independent apiary before core microbiome determination. Genera detected in ≥80% of apiaries and exhibiting a mean relative abundance ≥0.01% were classified as core taxa. For graphical representation, core genera reaching ≥1% mean relative abundance in at least one PFAS exposure-risk area were displayed individually, whereas lower-abundance core genera were grouped as “Other core genera” and genera not meeting the core microbiome criteria were grouped as “Non-core genera”.

For alpha-diversity analyses, including richness, Pielou’s evenness, and Shannon index, the count table was normalized using the Geometric Mean of Pairwise Ratios (GMPRs) method implemented in the GMPR package [80]. Differences in alpha-diversity metrics among groups were assessed using the non-parametric Kruskal–Wallis test. When significant differences were detected, pairwise Wilcoxon rank-sum tests were performed, and *p-*values were adjusted for multiple comparisons using the False Discovery Rate (FDR) method. Beta-diversity was evaluated using Bray–Curtis dissimilarity index and visualized via Non-metric Multidimensional Scaling (NMDS) ordination plots. Differences in microbial community composition among groups were tested using permutational multivariate analysis of variance (PERMANOVA). Statistical significance was set at *p*-value < 0.05. Differential abundance analysis was performed using the ANCOM-BC package [81], and effect sizes were reported as Log Fold Changes (LFCs) on the natural logarithmic scale. Positive LFC values indicated higher bias-corrected abundance in the red area relative to the brown area. Taxa with a prevalence below 10% were excluded prior to differential abundance analysis. Multiple-testing correction was performed using the Holm method, with statistical significance set at an adjusted *p*-value < 0.05. Pseudo-count sensitivity analysis was enabled to assess the robustness of differential abundance results.

### 2.6. Microbial-Association Network Analysis

An exploratory microbial-association network analysis was performed to investigate potential differences in the organization of genus-level bacterial associations among the Red, Yellow, and Brown areas. Networks were inferred separately for each area using the genus-level abundance table. Before network inference, rare taxa were minimally filtered, zero values were replaced using a pseudo-count approach, and abundance data were transformed using the Central Log-ratio (CLR) transformation. Area-specific association networks were constructed in R using the NetCoMi package [82], with Spearman’s correlation as the association measure. Network properties were calculated using the netAnalyze function and included edge density, percentage of positive associations, average dissimilarity, average path length, clustering coefficient, modularity, and natural connectivity. Topological properties were evaluated on the largest connected component of each network. Network modules were identified using the fast-greedy community detection algorithm, while taxa showing the highest eigenvector centrality were considered network hubs. To facilitate visualization, hub-centered subnetworks were generated, including the identified hub taxa and the genera directly connected to them. Given that only two biologically independent apiaries were included per PFAS exposure-risk area, the network analysis was considered exploratory and descriptive.

### 2.7. Statistical Analyses

To investigate whether the PFASs presence was associated with specific patterns of pesticide, pathogen, or microbial occurrence, an exploratory presence–absence analysis was performed at the apiary level, for the same six apiaries selected for metagenomics. PFASs, pesticides, pathogens, and bacterial genera were converted into binary variables (presence = 1, absence = 0). For bacterial genera, relative abundance values greater than zero were considered as presence.

Pairwise co-occurrence matrices were generated by comparing the presence of each PFAS with each detected pesticide, pathogen, and bacterial genus (hereafter referred to as features). For each PFAS-feature combination, the numbers of apiaries in which: (a) both were present, (b) only PFAS was present, (c) only the feature was present, or (d) neither was present, was calculated. The strength of co-occurrence was quantified using the Jaccard similarity index (ranging from 0 to 1, with higher values indicating greater relative co-occurrence) and the odds ratio, where values >1 indicate a positive association. Statistical significance of the association between each PFAS and feature was assessed using Fisher’s exact test, and *p*-values were adjusted for multiple comparisons using the Benjamini–Hochberg FDR correction. Results were visualized as co-occurrence heatmaps of pesticides, pathogens, and bacterial taxa. Because of the limited number of apiaries, no statistical inference was attempted, and results were interpreted as descriptive patterns.

To explore whether PFAS concentrations were associated with changes in bacterial community composition, quantitative analyses were performed using PFAS concentrations and the relative abundance of bacterial genera. For each apiary, the total PFAS burden was calculated as the sum of the concentrations of all detected PFASs. Pairwise associations between PFAS concentrations (both individual compounds and total PFAS burden) and bacterial relative abundances were evaluated using Spearman’s rank correlation coefficient (ρ), which was selected because it does not assume normality and is robust for small sample sizes. The obtained *p*-values were adjusted for multiple comparisons using the Benjamini–Hochberg FDR procedure. Correlation matrices were shown as heatmaps. Even in this case, given the exploratory nature of the study and the limited number of apiaries (*n* = 6), emphasis was placed on the magnitude and direction of the observed correlations rather than on statistical significance.

## 3. Results

### 3.1. On-Site Evaluation of Apiary Management and Hive-Health Assessment

The data obtained from observations collected through questionnaires during sampling showed that all apiaries were located in plain areas except for apiary L-VI-10, situated in the mountains. Concerning the territory, 13 out of the 15 apiaries were surrounded by agricultural land characterized by annual crops (grains and vegetables), as well as permanent croplands (orchards and vineyards). The remaining two apiaries (H-VI-2 and I-PD-3) were located in a rural area surrounded by a woodland and in a mixed agricultural and industrial area, respectively.

Regarding treatments against *Varroa* mite and *Vairimorpha* spp., in 14 out of the 15 apiaries, winter varroosis control was carried out using oxalic acid applied via sublimation, trickling, or extended-release strips. No veterinary drugs were used in the colonies during the sampling period or in previous months, except for the apiary H-VI-5, which was treated for *Varroa* mite using amitraz-based plastic strips (Apivar, Véto-pharma S.A., Palaiseau, France) five days before sample collection. Only in apiary I-PD-2 a *Vairimorpha* spp. treatment was carried out using a complementary feed based on medicinal herbs and B-group vitamins.

No abnormal behavior or clinical signs of infection were observed across the sampled apiaries. The colonies exhibited normal flight and foraging activities, with foragers actively collecting pollen. Queens were present in all hives during sampling, with years of birth ranging between 2022 and 2024; only in one apiary (I-PD-2) the queen’s age was unknown, due to the lack of marking.

Regarding bee nutrition, no supplementary feed was administered in four apiaries (H-VI-7, I-VI-8, I-PD-1, L-VR-1); however, the remaining eleven beekeepers provided a sugar solution or candy to support colony feeding.

### 3.2. Detection of PFAS Residues

The presence of 14 different PFASs in honey bee homogenates was investigated using LC–MS/MS method. In Table 1 the concentrations of PFAS measured in honey bees sampled in apiaries located in the three areas with varying levels of PFAS exposure risk are reported.

All the apiaries (*n* = 7) located in the Red area (high-risk area) resulted positive for at least one of the 14 target analytes. The short-chain PFASs were predominant, and all the sampled apiaries resulted positive for PFBA. The highest number of PFASs (*n* = 9) was detected in honey bees from apiary H-VI-1. These ones were represented by six of the investigated short-chain PFASs (PFBA, PFPeA, PFBS, PFHxA, PFHpA, and PFHxS), with PFBA and PFPeA exhibiting the highest concentrations (1.924 µg kg^−1^ and 1.025 µg kg^−1^, respectively). Among the long-chain PFASs, PFOA, PFNA, and PFUnA were detected, with PFOA reaching a maximum concentration of 3.03 µg kg^−1^; by contrast, PFOS, PFDA, and PFDoA were never detected. Notably, the quantitative levels of eight out of nine target compounds in apiary H-VI-1 were higher than those recorded across all other apiaries within the high-risk zone. H-VI-7 was the second most relevant apiary in terms of PFAS occurrence: it tested positive for three short-chain (PFBA, PFPeA, and PFBS) and two long-chain PFASs (PFOA and PFNA), with PFBA reaching a concentration of 2.69 µg kg^−1^. However, the honey bees from the remaining apiaries showed lower exposure profiles: H-VI-5 was positive for PFBA, PFOA, and PFNA; only two PFASs were detected in apiaries H-VI-2 and H-VI-4 (PFBA, PFPeA and PFBA, PFNA, respectively), whereas only PFBA was detected in apiaries H-VI-3 and H-VI-6 (0.104 µg kg^−1^ and 0.052 µg kg^−1^, respectively).

Regarding the four apiaries located in the Yellow area (intermediate-risk area), I-PD-3 resulted positive for both PFNA and PFBA (0.122 µg kg^−1^ and 0.09 µg kg^−1^, respectively); only PFNA was detected in I-PD-2 (0.163 µg kg^−1^), whereas honey bees from I-VI-8 and I-PD-1 were negative for all the 14 target PFASs.

Finally, target PFASs were undetectable in honey bees sampled within the Brown/Green area (low-risk area), except for L-PD-4, where only PFOS (a long-chain PFAS) was detected at a concentration of 0.087 µg kg^−1^.

Interestingly, the two short-chain PFASs GenX and C_6_O_4_, which are considered of increasing interest for their detection in rivers and drinking waters and the paucity or still-incomplete toxicological data, have never been detected in honey bees from all the apiaries considered in the present study [68,83].

### 3.3. Detection of Pesticide Residues

The analytical results concerning the presence of 292 pesticide residues in forager bees collected from apiaries located in high-, intermediate-, and low-risk areas of PFAS exposure, and analyzed using GC–MS/MS and LC–MS/MS *in-house* validated protocols, are summarized in Table 2.

Similarly to PFASs, the highest number of pesticide residues was found in honey bees sampled in five out of seven apiaries (H-VI-1, H-VI-2, H-VI-5, H-VI-6, H-VI-7) from the area of high-risk of exposure to PFASs (Red area). In particular, the simultaneous presence of two fungicides, cyazofamid and zoxamide, was detected in apiaries H-VI-1 (0.0037 µg/bee and 0.0016 µg/bee, respectively) and H-VI-2 (0.0012 ng/bee and 0.0010 µg/bee, respectively). The acaricide amitraz was detected in apiary H-VI-5 (0.0018 µg/bee), while chlorfenvinphos, a banned organophosphate insecticide and acaricide, was detected in apiaries H-H-VI-6 (0.0012 µg/bee) and H-VI-7 (0.0244 µg/bee).

On the other hand, no pesticide residues were detected in the four apiaries located in the area of intermediate-risk of PFAS exposure (Yellow area), whereas in the low-risk one (Brown area), only one out of four apiaries (L-VR-1) showed residues of the fungicides propamocarb (0.0015 µg/bee) and trifloxystrobin (0.0020 µg/bee).

Overall, pesticides residues were more frequently detected in apiaries from the high-risk PFAS exposure area, with concentrations ranging from 0.0010 to 0.0244 µg/bee. The highest concentration was recorded for chlorfenvinphos in apiary H-VI-7, located in the province of Vicenza.

### 3.4. Detection of Honey Bee Pathogens

The presence of eleven pathogens of different types, including fungi, protozoa, and viruses, were investigated by using biomolecular protocols (i.e., PCR, RT-PCR, and Real Time RT-PCR) set up and validated *in-house.* The results for each apiary are reported in Table 3.

During sampling, no apiary exhibited symptoms possibly associated with the presence of pathogens, such as aggressiveness, deformed wings, or trembling.

All the fifteen apiaries were negative for the protozoa *C. mellificae*, whereas four of them tested positive for *L. passim*, including one located in the high-risk zone for PFAS exposure (H-VI-7) and three in low-risk zones (L-VR-1, I-PD-4, and L-VI-9).

*V. ceranae* was detected in six apiaries (H-VI-3, H-VI-7, I-PD-2, I-PD-3, L-VR-1, L-PD-4), which were equally distributed across the three different PFAS exposure-risk zones.

Regardless of the PFAS exposure-risk zone, more than one honey bee virus was detected in each apiary, with various co-infections of two to five different viruses. Of the seven viruses investigated, DWV and SBV were detected in all the fifteen apiaries, whileBQCV was also widespread except in I-VI-8 (intermediate-risk area) and L-VR-1 (low-risk area). Six apiaries tested positive for CBPV, of which four were in the high-risk area (H-VI-1, H-VI-3, H-VI-4, H-VI-7), and two were in the low-risk area for PFAS exposure (L-VR-1, L-VI-9). ABPV was detected in two apiaries in the high-risk area (H-VI-4 and H-VI-6) and in one apiary in the low-risk of PFAS exposure area (L-VI-10). Finally, all the apiaries were negative for KBV and IAPV.

Overall, all the apiaries were tested negative for *V. apis*, *C. mellificae*, IAPV, and KBV.

### 3.5. Honey Bee Gut-Bacterial Community

#### 3.5.1. Metataxonomic Results

A total of 11,595,085 raw-reads (mean per sample = 644,171.4) were obtained from *16S rRNA* gene sequencing. After quality filtering, 2604 unique ASVs were amenable for comparative analysis. With respect to taxonomic composition, 25 bacterial genera were identified as members of the study-specific core microbiome. In Figure 2, core genera reaching ≥1% mean relative abundance in at least one exposure-risk area are displayed individually, whereas lower-abundance core genera are grouped as “Other core genera” and non-core genera as “Non-core genera”. The complete genus-level composition and core/non-core classification are provided in Supplementary Table S5. The overall microbial community was largely composed of bacterial taxa belonging to the genera *Lactobacillus*, *Bartonella*, *Bombilactobacillus*, *Snodgrassella*, *Gilliamella*, *Bifidobacterium*, and *Gluconacetobacter*. Moreover, these taxa represented approximately 80% of the entire microbial composition inferred from the dataset analyzed here. The remaining 20% of bacterial genera included a mixture of core bee-gut-associated symbionts (e.g., *Apilactobacillus*, *Bombella*, *Fructobacillus*, and *Leuconostoc*) [84,85], environment-derived taxa (e.g., *Gluconobacter*, *Asaia*) [85,86] and opportunistic bacteria such as *Enterobacter*, *Klebsiella*, *Serratia*, *Citrobacter*, *Raoultella*, *Pantoea*, *Acinetobacter*, *Escherichia*, *Morganella*, and *Hafnia* [48,87,88]. These taxa are commonly found in diverse habitats and may represent transient colonizers of the bee gut.

Regarding microbial community diversity, the gut microbiota of bees sampled from areas with different levels of PFAS contamination was assessed using alpha-diversity analyses. The richness index (Figure 3A), defined as the number of distinct ASVs identified across samples, showed an increasing trend from the Brown to Yellow and Red areas. Significant differences were observed between Brown and Red areas as well as between Yellow vs. Red areas; however, no significant difference was detected in Brown vs. Yellow areas. By contrast, both Pielou’s evenness index and Shannon diversity index (Figure 3B,C) remained stable across the three areas, with no statistically significant differences. Concerning beta-diversity (Figure 3D), the Non-metric Multidimensional Scaling (NMDS) plot combined with PERMANOVA analysis revealed a significant difference in gut-microbiota composition between honey bees collected in the Brown area and those in the Red area (pseudo-F_2,15_ = 2.39, R^2^ = 0.242, *p*-value = 0.001).

ANCOM-BC analysis identified significant differences in the relative abundances of *Lelliottia* (LFC = 2.82; adjusted *p* = 0.030) and *Klebsiella* (LFC = 5.63; adjusted *p* = 0.0014), both of which were more abundant in the Red than in the Brown area (Figure 4). Although *Lelliottia* was characterized by a low relative abundance, its mean abundance increased from 0.0048% in the Brown area to 0.129% in the Yellow area and 0.441% in the Red area.

#### 3.5.2. Microbial Association Network Structure

Exploratory genus-level association networks showed descriptive area-specific differences in topology and hub composition (Table 4, Figure 5). The Red area network had the highest edge density (0.620), proportion of positive associations (63.18%), and natural connectivity (0.266); moreover, it was characterized by several central genera, including *Legionella*, *Silvania*, *Streptomyces*, *Vagococcus*, and *Pluralibacter*. Conversely, the Yellow area network showed the highest clustering coefficient and modularity (0.750 and 0.161, respectively), indicating a comparatively more compartmentalized structure organized around *Sinocapsa* spp., *Sinorhizobium* spp., *Dulcicalothrix* spp., and *Paludibacter* spp. Finally, the Brown area network displayed lower density (0.566), clustering coefficient (0.708), and natural connectivity (0.216), and was mainly organized around *Solirubrobacter* spp. and *Paraclostridium* spp. As a whole, these results suggest descriptive differences in the organization of bacterial associations among the PFAS exposure-risk areas; however, these patterns are reported descriptively only and should be regarded as preliminary because each exposure-risk area was represented by only two biologically independent apiaries.

### 3.6. PFAS–Feature Co-Occurrence Patterns Across Apiaries

Qualitative co-occurrence analyses between PFAS and pesticides, pathogens, and bacterial genera were performed across the six investigated apiaries using Jaccard similarity and odds ratios (Tables S6–S8; Figure S1A–C). Overall, the analyses revealed sparse and heterogeneous patterns of PFAS–feature co-occurrence. Although several pairs showed high Jaccard indices or infinite odds ratios, these estimates were largely driven by the small number of apiaries and the low prevalence of several features. None of the associations was statistically significant after Fisher’s exact test and Benjamini–Hochberg false discovery rate (FDR) correction (Table S6). PFAS–pesticide co-occurrences were sporadic and did not support a specific relationship between these two variables. Similarly, pathogens displayed a heterogeneous presence–absence profile across apiaries, with no consistent clustering according to PFAS status. PFAS-positive apiaries did not exhibit a distinct pathogen profile compared with PFAS-negative apiaries, suggesting that the observed overlaps were more likely related to the sparse distribution of PFAS and pathogen detections than to a specific association between the two categories.

Among the bacterial genera, the highest apparent co-occurrence was observed for the PFNA-*Microbacterium* pair, which was detected in three apiaries and absent in the remaining three, thus resulting in a Jaccard index of 1.00 and an infinite odds ratio. PFPeA, PFBS, and PFOA also showed complete co-occurrence with the genera *Citrobacter*, *Escherichia*, *Microvirga*, *Paenibacillus*, *Parasynechococcus*, *Priestia*, and *Raoultella* in two apiaries (H-VI-1 and H-VI-7) for each PFAS, yielding Jaccard indices of 1.00 and infinite odds ratios. When the analysis was restricted to members of the honey bee core microbiota, PFBA and PFNA co-occurred with *Bifidobacterium*, *Bombilactobacillus*, *Gilliamella*, *Lactobacillus*, and *Snodgrassella* in three apiaries, corresponding to Jaccard indices of 0.50 (H-VI-1, H-VI-7 and I-PD-3 for PFBA; H-VI-1, I-PD-2, and I-PD-3 for PFNA). PFPeA, PFBS, and PFOA co-occurred with the same core-associated taxa in two apiaries (H-VI-1 and H-VI-7), with Jaccard indices of 0.33 (Table S6). These patterns should be interpreted as descriptive rather than as evidence of specific PFAS-associated shifts in bacterial occurrence.

Because the presence–absence analyses did not find changes in relative microbial abundances, quantitative associations between PFAS concentrations and bacterial genera were subsequently assessed using the Spearman’s correlation analysis (Tables S7 and S8; Figure 6). Across all detected genera, the correlation matrix showed a broad and heterogeneous range of coefficients. The strongest positive coefficients reached ρ = 1.00 for PFPeA, PFBS, and PFOA in association with *Citrobacter*, *Escherichia*, *Microvirga*, *Paenibacillus*, *Parasynechococcus*, *Priestia*, *Raoultella*, and *Silvania*; likewise, for PFHxA, PFHpA, PFHxS, and PFUnA in association with several low-prevalence genera, including *Pseudocitrobacter*, *Buttiauxella*, *Tanticharoenia*, *Romboutsia*, *Agrobacterium*, *Chthoniobacter*, *Clostridium*, *Pseudescherichia*, *Arcobacter*, *Brevibacterium*, *Desertimonas*, *Angustibacter*, *Chlamydia*, *Micromonospora*, *Nocardiopsis*, *Sandaracinus*, *Simiduia*, *Thiomicrorhabdus*, and *Virgibacillus*. Within the core microbiota, the strongest positive correlation was observed between PFOS and *Lactobacillus* spp. (ρ = 0.65). However, the strongest negative correlation involved the genera *Frischella*, *Gluconacetobacter*, and *Potamosiphon* with PFPeA, PFBS, and PFOA (ρ = −0.85); additional negative correlations were observed between *Arsenophonus* and *Pantoea* with PFNA (ρ = −0.76) and, finally, *Frischella* and *Gilliamella* with PFHxA, PFHpA, and PFHxS (ρ = −0.65). However, none of the quantitative associations remained significant after FDR correction.

## 4. Discussion

The constant interaction between honey bees and their surrounding ecosystem continuously exposes them to both abiotic and biotic stressors, which also include environmental pollutants and pathogens, respectively. It is a well-established concept that pollinators (including honey bees) are constantly exposed to anthropogenic agrochemicals and pollutants dispersed in the air, soil, and water. Given their usefulness to ascertain in situ, in vivo, as well as under laboratory conditions (e.g., by using OECD-approved acute and chronic tests) and the effects of xenobiotics on their homeostasis, honey bees were, and still are, ideal and sophisticated bioindicators of environmental contamination; moreover, they are receiving increasing attention in comparative toxicological risk assessment [8].

On a toxicological comparative evaluation, the impact on honey bee health of pesticides (e.g., insecticides, acaricides, fungicides, herbicides, fertilizers) and classical environmental contaminants (e.g., heavy metals, dioxins, or dioxin-like compounds) has been the subject of extensive investigations; however, limited information are available on the effects of “emerging contaminants”, such as mycotoxins, microplastics, rare earth elements, human drugs (e.g., carbamazepine) and, more recently, PFASs [37,89,90,91,92,93]. PFASs are termed “forever chemicals” due to their environmental persistence, widespread distribution, and potential toxicity [17]. In the last five years, environmentally relevant concentrations of PFOS (a legacy and long-chain PFAS) have been shown to affect honey bee colony activity, hive maintenance, and defense [4]; moreover, PFOA and PFHxS (a legacy long-chain PFAS and a short-chain alternative to PFOS, respectively) have been shown to substantially affect honey bee gut health [65]. All this scientific evidence highlights the importance of investigating PFASs in bees and hive products [37].

The results of our study on the presence of PFASs across areas with varying exposure-risks in the Veneto Region further support the role of the honey bee as an effective environmental bioindicator, as already extensively demonstrated in the literature, particularly for other environmental pollutants, such as pesticides and heavy metals [6,13,33,91,94,95]. Furthermore, our findings indicate a clear gradient effect in PFAS contamination: apiaries located within the high- PFAS exposure-risk area (i.e., the Red area) showed a contamination profile characterized by the highest levels of PFAS bioaccumulation, despite the concurrent occurrence of some pesticide residues and common pathogens.

When comparing the honey bees sampled across the three investigated areas, the highest number of PFASs, occurring in different combinations and for some of them at concentrations higher (>1 µg kg^−1^) than the levels detected in surface waters, was observed in apiaries within the Red area. In contrast, in the intermediate- (Yellow area) and low-risk (Brown area) areas, PFASs were never detected, or the positivity was restricted to PFNA (0.122 µg kg^−1^) and PFBA (0.09 µg kg^−1^) in a single apiary in the intermediate-risk area, and to PFNA in one apiary each within the intermediate- (0.163 µg kg^−1^) and low-risk (0.087 µg kg^−1^) areas. 

Among honey bees sampled in the high-risk area, the predominant PFASs were short-chain compounds (PFBA, PFPeA, PFBS, PFHxA, PFHpA, and PFHxS), with PFBA detected in all samples.

The detection of short-chain PFASs and particularly of PFBA, which peaked at 1.924 µg kg^−1^ and 2.69 µg kg^−1^ in apiaries H-VI-1 and H-VI-7 respectively, corroborates the current ecotoxicological trends reporting the high environmental mobility and water-solubility of these shorter compounds [37,96,97,98]. Indeed, while long-chain PFASs mostly bioaccumulate in trophic webs owing to their lipophilic nature, short-chain compounds are highly bioavailable in surface waters, soil pore water, and nectar; therefore, they could result in a rapid uptake by foraging bees. As a consequence, short-chain PFASs can be detected at quantifiable levels not only in foragers, hive bees, and pollen [37], but also in honey [99]. With regard to long-chain PFASs (PFOA, PFNA, PFOS, and PFUnA), the highest concentrations of PFOA and PFNA were detected in honey bees from apiary H-VI-1 (3.03 µg kg^−1^ and 0.155 µg kg^−1^, respectively). When comparing the PFAS concentrations detected in honey bees with those reported in surface waters by ARPAV monitoring stations (WebGIS platform; at https://www.arpa.veneto.it—accessed on 20 August 2026) [100], a similar pattern was observed in terms of the PFAS congeners detected; however, for some of them the concentrations found in honey bees were higher than those recorded in water samples. A possible and reasonable explanation for such a bioaccumulation is that PFAS uptake, as happens with other pollutants, may derive not only from water and food sources, but also from air or direct contact, leading to increased xenobiotic concentrations within the body. Given the short lifespan of honey bees, these high concentrations do not reflect long-term PFAS exposure and, consequently, bioaccumulation; rather, they may provide a real-time indicator of xenobiotic environmental pressure on biota that could be underestimated by water sampling alone, further supporting the concept that these insects are highly sensitive bioindicators reflecting spatial and temporal pollution trends.

In contrast to the evidence available for other animal species and humans [27,101,102], information on the biological effects and bioaccumulation of many PFASs on *A. mellifera* or other pollinators is still limited. PFOS remains the most extensively studied PFAS in honey bees [4,65,66], whereas data on PFOA [64,65] and PFHxS [65] have only recently become available. Regarding PFOS, the study by Sonter et al. (2021) [4] demonstrated that exposure to environmental concentrations (0.02–1.6 mg L^−1^) induces a significant increase in honey bee mortality and inhibits brood development at concentrations equal to or greater than 0.8 mg L^−1^ and 0.02 mg L^−1^, respectively. Exposure to sublethal PFOS concentrations (5 to 20 μg L^−1^ in sugar syrup) resulted in bioaccumulation in bee tissues, its detection in feces, and its transfer to honey across all tested concentrations; furthermore, adverse life stage development and intergenerational effects were evident even at the lowest exposure concentration of 5 μg L^−1^ [66]. As demonstrated by Lei et al. (2025) [64], PFOA exerts toxic effects in *Apis cerana* through diverse mechanisms, including intestinal injury, immune suppression and, interestingly, microbiome dysbiosis. Similar effects were observed in *A. mellifera* exposed to environmentally relevant concentrations (0.5–50 μg L^−1^) of PFOA and PFHxS, resulting in compromised gut health characterized by midgut epithelial damage, disrupted barrier integrity, altered glycerophospholipid metabolism, and the activation of inflammatory and apoptotic pathways [65]. Moreover, the toxicological impact of PFHxS was more pronounced than that of PFOA.

Compared with PFAS, the impact of anthropogenic agricultural practices (i.e., the use of plant protection products, PPPs) on honey bee physiology, foraging, colony, and ultimately survival has been extensively investigated. Pesticides are a broad spectrum of agrochemicals widely deployed to protect crops from insect pests, weeds, and fungi, to maximize yields. Overall, pesticides can be classified into three main categories: herbicides, insecticides, and fungicides. According to their mode of action, they can be classified as destructive, repellent, and mitigating PPPs. The extensive use of pesticides may result in both acute-lethal and chronic sub-lethal effects on honey bees, ultimately compromising their individual health, colony stability, and overall population dynamics, as recently reviewed by Musah et al. (2026) [103]. In our study, the presence of more than 292 pesticide residues was investigated in forager bees collected from high-, intermediate-, and low-risk areas of PFAS exposure, by using GC–MS/MS and LC–MS/MS. Overall, pesticide residues were detected in six out of the fifteen apiaries investigated, with a higher frequency of occurrence in the high-risk of PFAS exposure area. The presence of such pesticide residues in honey bees collected in this area may be related to more intensive land use, such as orchards and vineyards, compared with the other two areas. More specifically, two acaricides/insecticides (chlorfenvinphos and amitraz), and four fungicides (cyazofamid, zoxamide, propamocarb, and trifloxystrobin) were detected. However, their concentrations were low, ranging from 0.0010 to 0.0244 µg/bee. The organophosphate insecticide chlorfenvinphos, an acetylcholinesterase inhibitor considered a concern for bees (https://sitem.herts.ac.uk/aeru/ppdb/en/—accessed on 20 August 2026) [104], was detected at the highest concentration in apiary H-VI-7 (0.0244 µg/bee; Red area). Therefore, its potential contribution to PFAS-related toxicity in honey bees could not be excluded a priori, drawing on the well-known concept of the toxic effects of mixtures of environmental contaminants on biota, as already hypothesized for humans [105]. Worth noting, chlorfenvinphos is currently not approved as an active substance under Regulation (EC) No 1107/2009 [106] and its unauthorized or illegal use cannot be excluded. Conversely, the other detected pesticides, i.e., cyazofamid, zoxamide, propamocarb, trifloxystrobin, and amitraz, are currently approved for use within the European Union. These compounds are generally characterized by low acute toxicity to honey bees if compared with insecticides (Table S4), and the detected concentrations were several orders of magnitude lower than the reported acute toxicity thresholds. Nevertheless, these substances should not be considered entirely harmless to pollinators; chronic exposure and interactions with other xenobiotics may alter forager bee behavior, detoxification pathways, immunity, again gut microbiota, and colony performance [107,108].

Apart from anthropogenic compounds, honey bees face multiple pathogenic threats, including parasitic mites, viruses, and bacterial diseases, such as *V. destructor*, DWV, and *Paenibacillus larvae*, respectively [109,110]. Under our sampling conditions, honey bees from the different apiaries did not exhibit clinical symptoms attributable to pathogen infection, such as deformed wings, trembling, uncoordinated movements, reduced body size, dark and hairless individuals, or mortality around the hives. However, biomolecular analyses detected the presence of common honey bee pathogens in all fifteen apiaries, although a high degree of variability in the number and combination of pathogens was observed, with co-infections ranging from two to six different pathogens. Notably, the statistical analysis demonstrated that the distribution of bee pathogens was not significantly correlated with the presence of PFAS in the three study areas.

As mentioned above for xenobiotics, a healthy gut microbiota provides protection against honey bee pathogens, including bacteria, fungi, and viruses. Several studies have demonstrated that pathogen infections, by disrupting or dysregulating the gut microbiota, may result in changes in microbial community structure and altered expression of genes involved in immunity, metabolism, behavior, and development [48,49]. Interestingly, *V. ceranae* (detected in six apiaries, two per each risk area), *L. passim* (found in one apiary in Red area, and in three apiaries of Brown area), and DWV and SBV viruses (commonly detected in all apiaries), are likely to induce alteration in honey bee gut microbiota [49,56,58,111,112,113,114].

As briefly mentioned above, and from a comparative perspective, the gut microbiota plays a crucial role in regulating host development and a wide range of biomolecular processes, including metabolism, immune function, growth, and development. In addition, the microbiome is well recognized to influence central nervous system function and behavioral processes in mammals, through several pathways generally referred to as the gut–brain axis. In this context, insects have emerged as valuable comparative animal models for investigating host–microbiota interactions. Indeed, many insects harbor simplified and more specialized microbial communities, providing a reductionist advantage for mechanistic studies. Among social insects, honey bees represent an exceptional animal model [115,116]. However, beyond their potential use in comparative mechanistic studies on host–microbiota interactions, recent published studies have also underscored how honey bee health is closely and intrinsically linked to a healthy and stable gut microbiota, which plays a vital role in preserving the general health of honey bees and their colonies [48,117]. Honey bees are frequently exposed to abiotic and biotic factors that can alter their gut microbiota and, consequently, affect their biology and health. The most common ones are pathogens (bacteria, fungi, viruses), beekeeping and agricultural practices, such as the use of antibiotics (e.g., tetracyclines, tylosin) and pesticides, including insecticides (e.g., neonicotinoids), fungicides (e.g., chlorothalonil), and herbicides (e.g., glyphosate). Other anthropogenic activities may also contribute to the release of contaminants and pollutants, such as PFASs, phthalates, dioxins, and dioxin-like compounds into the environment [49]. Honey bees are widely recognized as effective bioindicators of environmental health and may therefore be potentially useful for environmental-risk assessments [8]. Building on this concept, recent metagenomic studies across different landscapes have suggested that the honey bee gut microbiota may also serve as an indicator of environmental contamination, given its sensitivity to pesticides and other chemical stressors [61,118,119,120].

In the present study, we demonstrated that PFASs can bioaccumulate in honey bees, albeit in the co-presence of some pesticides and common pathogens. To date, only a few studies have investigated the effects of PFASs on this key managed pollinator species; moreover, the potential modulation of the bee gut microbiota following PFAS exposure remains largely unexplored. Therefore, the second objective of our study was to investigate the impact of PFASs on honey bee gut microbiota composition, by comparing apiaries selected from three areas characterized by different levels of PFAS environmental contamination, using a metagenomic approach.

In all honey bee samples, the microbiota composition was largely dominated by *Lactobacillus* spp., *Bartonella* spp., *Bombilactobacillus* spp., *Snodgrassella* spp., *Gilliamella* spp., *Bifidobacterium* spp., and *Gluconacetobacter* spp., accounting for approximately 80% of the total bacterial composition. This indicates that the canonical “core” microbiota, composed of a small set of the aforementioned genera, was largely preserved despite the different environmental contexts, and consistent with the idea that the honey bee microbiota is relatively conserved [49]. Alpha- and beta-diversity analyses, used to assess microbiota richness and diversity within (alpha-diversity) and between (beta-diversity) the PFAS exposure areas, revealed an increasing trend in the richness index (number of distinct ASVs detected) from the Brown (low-risk) to the Yellow (intermediate-risk) and Red (high-risk) areas, while no significant differences were observed between the Brown (low-risk) and Yellow (intermediate-risk) areas. The beta-diversity analysis showed a significant difference in honey bee gut community composition between the Brown (low-risk) and Red (high-risk) areas. At the taxonomic level, ANCOM-BC analysis showed statistically significant differences in the relative abundances of *Klebsiella* spp. and *Lelliottia* spp., which resulted significantly enriched in samples from the Red area (high-risk) compared to those from the Brown (low-risk) area. These two Gram-negative bacteria from the *Enterobacteriaceae* family have been detected in several insect species, including honey bees, *Bombyx mori*, and *Bactrocera dorsalis* [121,122]. Although their ecological role is not fully understood, members of these genera are generally considered opportunistic rather than stable members of the honey bee core microbiota, inhabiting freshwater environments including surface water, sewage, and soil, and often related to antimicrobial resistance [123]. The use of antibiotics in beekeeping practices, although discouraged, is permitted in the European Union according to Regulation (EU) 2019/6 [124], while they are extensively used in human and veterinary medicine. Therefore, their increased relative abundance may reflect either exposure to anthropogenically contaminated habitats or environmental contamination rather than being a direct consequence of PFAS exposure. The enrichment of *Lelliottia* should nevertheless be interpreted with caution because this genus occurred at low relative abundance (<1% across all exposure areas). Although its abundance showed a consistent increase from the Brown to the Red area and the differential abundance result remained significant after multiple-testing correction and passed the ANCOM-BC sensitivity analysis, this finding should therefore be regarded as exploratory and requires confirmation in larger datasets.

The co-occurrence network analysis provided further insight into the influence of environmental conditions on the ecological organization of honey bee gut bacterial communities. The PFAS high-risk exposure area network (Red) was the most densely connected, and was characterized by hub taxa such as *Legionella*, *Silvania*, *Vagococcus*, *Pluralibacter,* and *Streptomyces*. This pattern suggests that the bacterial community in these apiaries was structured around a limited number of highly connected taxa. Such an organization may reflect the influence of heterogeneous environmental inputs or stressors affecting the apiaries. Some of the aforementioned genera are associated either with environmental habitats or with microorganisms commonly found outside the honey bee gut. As an example, *Legionella* is widely distributed in aquatic environments and can occur within biofilm-associated microbial communities [125], whereas *Silvania* has been isolated from industrial effluents and has been investigated for its potential to degrade environmental contaminants [126]; *Vagococcus* is usually isolated from fish and domestic animals [127], while *Pluralibacter* has been reported in environmental and clinical contexts [128]. The Yellow-area network showed the highest degree of modularity among the three investigated areas, indicating a more compartmentalized microbial structure. Nevertheless, recognizable bee-associated taxa remained embedded within a network also including environmentally associated genera, such as *Sinocapsa*, *Sinorhizobium*, *Dulcicalothrix*, and *Paludibacterium*. While *Sinocapsa* and *Dulcicalothrix* are cyanobacterial genera often found in fresh water and terrestrial habitats, *Sinorhizobium* is primarily associated with soil and plant roots, where it can establish symbiotic interactions with leguminous plants [129]. *Paludibacterium* has also been isolated from wetland soils and other aquatic or soil-associated environments [130]. This pattern may indicate a mixed community, in which the core gut microbiota remains relatively stable while additional microorganisms are introduced from environmental sources, including vegetation, soil, or water. Thus, the intermediate PFAS exposure-risk area network (Yellow) would represent a distinct community configuration rather than an intermediate state along a Red–Yellow–Brown area gradient. By contrast, the Brown-area network was characterized by a smaller number of highly connected genera, with *Solirubrobacter* and *Paraclostridium* emerging as the main hubs. This suggests a different pattern of taxon centrality and community organization compared with the other two PFAS exposure-risk areas. *Solirubrobacter* is commonly associated with rhizosphere and has been reported to be widely distributed across soil microbial communities [131]. *Paraclostridium* comprises bacteria isolated from soil, marine habitats, polluted waters and, occasionally, the human-intestinal microbiota [132]. Their ability to grow on a range of organic substrates and their occurrence in diverse environmental habitats suggest that the prominence of *Paraclostridium* in the Brown area network may be related to environmental inputs or to the availability of specific organic substrates around the sampled apiaries. Overall, the core microbiota remained largely conserved across the three areas, indicating that the main bee-associated bacterial taxa were not substantially replaced. Rather, the differences were mainly related to the ecological organization of the communities, particularly their co-occurrence patterns, modularity, and distribution of central taxa. The distinct hub genera identified in each network may therefore reflect differences in the environmental conditions surrounding the apiaries, including the type and intensity of contamination or pollution, anthropogenic practices, habitat structure and related alterations, availability and changes of specific crops or floral resources, as well as pathogen exposure. In this respect, the identification of *Streptomyces* as a hub is compatible with its broad environmental distribution and its occurrence in soil-associated microbial communities; such an interpretation is also consistent with the foraging behavior of *A. mellifera*, which may expose bees to microorganisms originating from plants, soil, water, and other environmental substrates. Notably, PFAS contamination may be part of the aforementioned environmental disturbances and may co-occur with other pollutants (e.g., mixtures), with altered land use and/or poorer resource quality. However, these network-derived observations must be interpreted with caution. Because only two biologically independent apiaries were available for each exposure-risk area, differences in edge density, modularity, centrality, and putative hub taxa cannot be considered stable ecological properties of the respective areas. The observed patterns may reflect local environmental conditions, biological variability among apiaries, or sampling variability and should therefore be considered preliminary and hypothesis-generating. Validation in a larger number of biologically independent apiaries will be required before robust ecological conclusions can be drawn from network topology.

To date, limited studies have investigated the relationship between PFAS exposure and microbiota dysbiosis in insects; nevertheless, an experimental study conducted on *Rana nigromaculata* reported that PFOA, PFOS, and 6:2 Cl-PFESA were associated with the animal’s microbiome, showing a higher number of co-occurrence network edges than controls [133]. Thus, our findings would support the hypothesis that PFAS exposure, or otherwise anthropogenically influenced environments, may partially affect bee gut microbiota; rather, they shift its organization and modestly influence its network structure. In such a context, the honey bee gut microbiota appears to be resilient at the level of its core members, yet sensitive enough to reflect broader ecological and chemical stress through changes in richness, taxonomic balance, and microbial connectivity. Clearly, the limited number of sampling units (only two apiaries per risk area) represents an important limitation of our study and precludes a generalization of the observed network patterns. Therefore, our results should be considered exploratory and interpreted primarily within the context of the specific apiaries investigated.

The combined qualitative and quantitative analyses revealed several apparent PFAS–bacteria associations. In particular, the presence–absence analyses identified numerous PFAS–bacteria co-occurrences, particularly involving PFBA and PFNA. Nevertheless, the highest Jaccard values and infinite odds ratios obtained were mostly associated with a low prevalence of individual PFAS and bacterial features rather than with a robust and independently supported relationship. Moreover, apparently strong associations involving PFAS, *Enterobacterales,* and environmental genera were based on only two or three co-occurring bee samples; therefore, they are more likely to reflect shared environmental sources, exposure conditions, or differences in colony-level ecological status. Finally, quantitative analyses showed that many of the qualitative associations were likely explained by the high prevalence of several core bacterial taxa rather than by changes in their abundances; moreover, they were unable to establish causality or distinguish a direct PFAS effect among the many confounding factors such as sampling location, season, colony condition, diet, or other environmental variables. This highlights the importance of integrating occurrence and abundance data when assessing relationships between environmental contaminants and host-associated microbiota, as the presence–absence analysis may overestimate ecological associations when dominant taxa are widely distributed among samples. Consequently, even in this case these findings should be considered exploratory and hypothesis-generating rather than confirmatory.

The absence of a clear distinction in the statistical co-occurrence patterns observed between PFAS-positive and PFAS-negative apiaries, together with the overall stability of the honey bee gut core microbiota, suggests that PFAS bioaccumulation in bees is unlikely to be associated with major shifts in bacterial community composition. However, the recurrent negative correlations observed for *Frischella* spp. and *Gilliamella* spp. would suggest that some members of the bee gut microbiota are more sensitive to PFAS exposure than the bacterial community as a whole. However, the lack of significant *p*-values, and the absence of FDR-significant associations, provide no statistical support for such a specific PFAS-associated microbiota signature. In addition, the limited number of apiaries, the large number of pairwise comparisons and the high prevalence of rare taxa reduce the reliability of effect-size estimates and increase the risk of unstable or spurious associations.

Overall, the results obtained in the present study should be regarded as hypothesis-generating rather than as evidence of causal relationships. Larger studies incorporating colony-level metadata and a wider environmental exposure gradient are needed to determine whether the observed patterns represent reproducible ecological relationships or primarily reflect sparse sampling and shared environmental exposure. Multivariable and compositional-data approaches, together with metagenomic or transcriptomic functional analyses (i.e., multi-omics approaches), will also be important for assessing whether PFASs affect the structure or functionality of the honey bee gut microbiota.

The present study has both strengths and limitations. Among the strengths, (a) based on the availability of beekeepers, the PFAS exposure-risk areas were identified according to the results of the ARPAV monitoring plan, ensuring field-realistic exposure conditions; (b) honey bees are considered an effective bioindicator of environmental health; however, very few studies have assessed the effects of different PFASs on this sentinel species, e.g., bioaccumulation and their possible transfer to hive products; (c) the effects of anthropogenic compounds on honey bee gut microbiota are gaining increasing interest among environmental toxicologists, also for its value as an indirect biomarker of exposure. To the best of our knowledge, our study is one of the few to investigate the effects of PFASs on honey bee gut-bacterial community; (d) although the honey bee gut microbiota has been extensively characterized in recent years, our metagenomic results will contribute to improve the knowledge about gut-microbiota composition and its possible variations in response to biotic and abiotic stressors, with a focus on PFASs; (e) overall, our results will provide new information relevant to PFAS environmental-risk assessment in the Veneto Region, where different PFAS contamination zones have been identified. The modest sample size suggests caution regarding the statistical power and the broader applicability of our findings (e.g., regarding causality vs. correlation of PFAS–microbiota composition relationships). Additionally, since the apiaries are kept under field-realistic conditions, they are continuously exposed to biotic stressors, such as pathogens (e.g., *V. ceranae*, DWV), pesticides commonly used in agricultural practices (e.g., neonicotinoid insecticides), and other environmental factors. Such co-presence makes it more complex to define the specific impacts of PFASs on the honey bee gut microbiota and to interpret the related statistical associations. However, these factors did not preclude us from assessing whether different PFASs are bioaccumulated in honey bees sampled in different PFAS exposure-risk areas and, most importantly, confirm the higher bioaccumulation of both legacy and alternative PFASs in honey bees sampled in the Red area (higher risk of PFAS exposure).

## 5. Conclusions

The present study highlights the relevance of honey bees as sensitive bioindicators for monitoring environmental PFAS contamination. PFASs were detected in honey bees, and their concentrations and chemical profiles varied according to the geographical location of the apiaries (high-, intermediate-, and low-risk of PFAS exposure), suggesting differences in local PFAS exposure patterns. Therefore, honey bees provide a valuable spatial and temporal overview of environmental pollution, supporting the development of environmental health maps and large-scale monitoring strategies. In addition, our results provide preliminary information about the effects of PFASs on honey bee gut community composition, although the co-occurrence, even if at low levels, of pesticides and pathogens, contributed to the lack of unequivocal evidence.

Although further investigations are required to clarify the mechanisms underlying PFAS-associated toxic effects, including potential alterations in the honey bee gut microbiota and their consequences for bee health, the use of honey bees as environmental sentinels represents a promising approach for assessing chemical pressure on ecosystems. The widespread occurrence of PFASs represents an emerging concern for biodiversity and ecosystem health, with potential implications extending beyond individual organisms and contributing to broader One Health concerns. In this context, biomonitoring approaches based on honey bees may support the identification of areas with different contamination pressures, contributing to future ecological-risk assessments and conservation strategies. In addition, OECD-related acute or chronic toxicity studies on single PFASs or their mixtures will help us understand the mechanistic toxicology of these emerging contaminants, and their potential molecular effects on mammals and particularly humans. This aspect will be the subject of future planned studies.

## Figures and Tables

**Figure 1:**
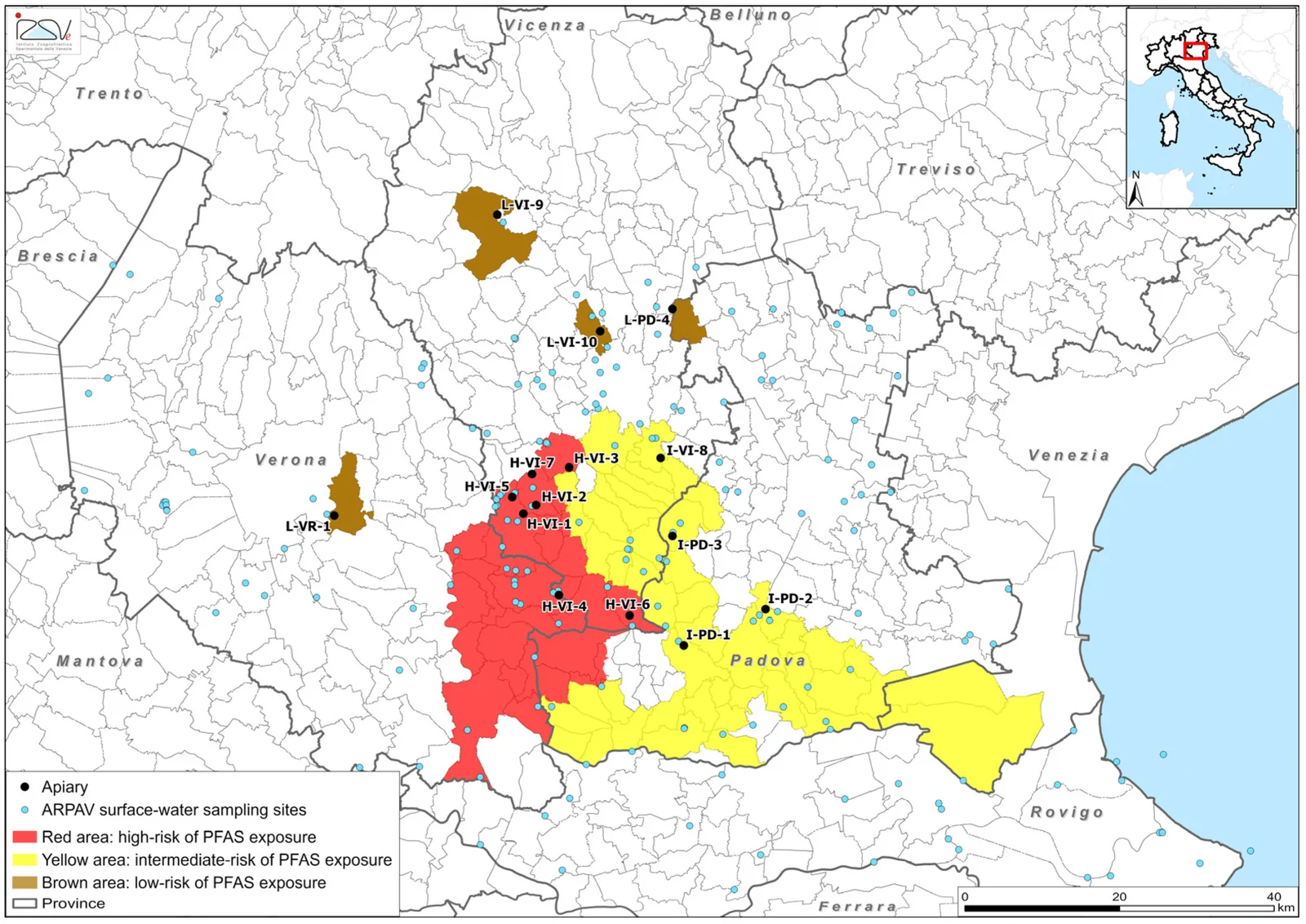
Veneto region (north-eastern Italy): distribution of the apiaries involved in the study. Each apiary is identified by an alphanumeric code consisting of the PFAS exposure risk level (H for High, I for Intermediate, L for Low), the province abbreviation (VI for Vicenza, PD for Padova, VR for Verona) in which it is located followed by a progressive number (e.g., H-VI-1, I-PD-1, L-VR-1, etc.). Colored areas represent the different levels of PFAS exposure risk, based on data from the water monitoring program available on the ARPAV website.

**Figure 2:**
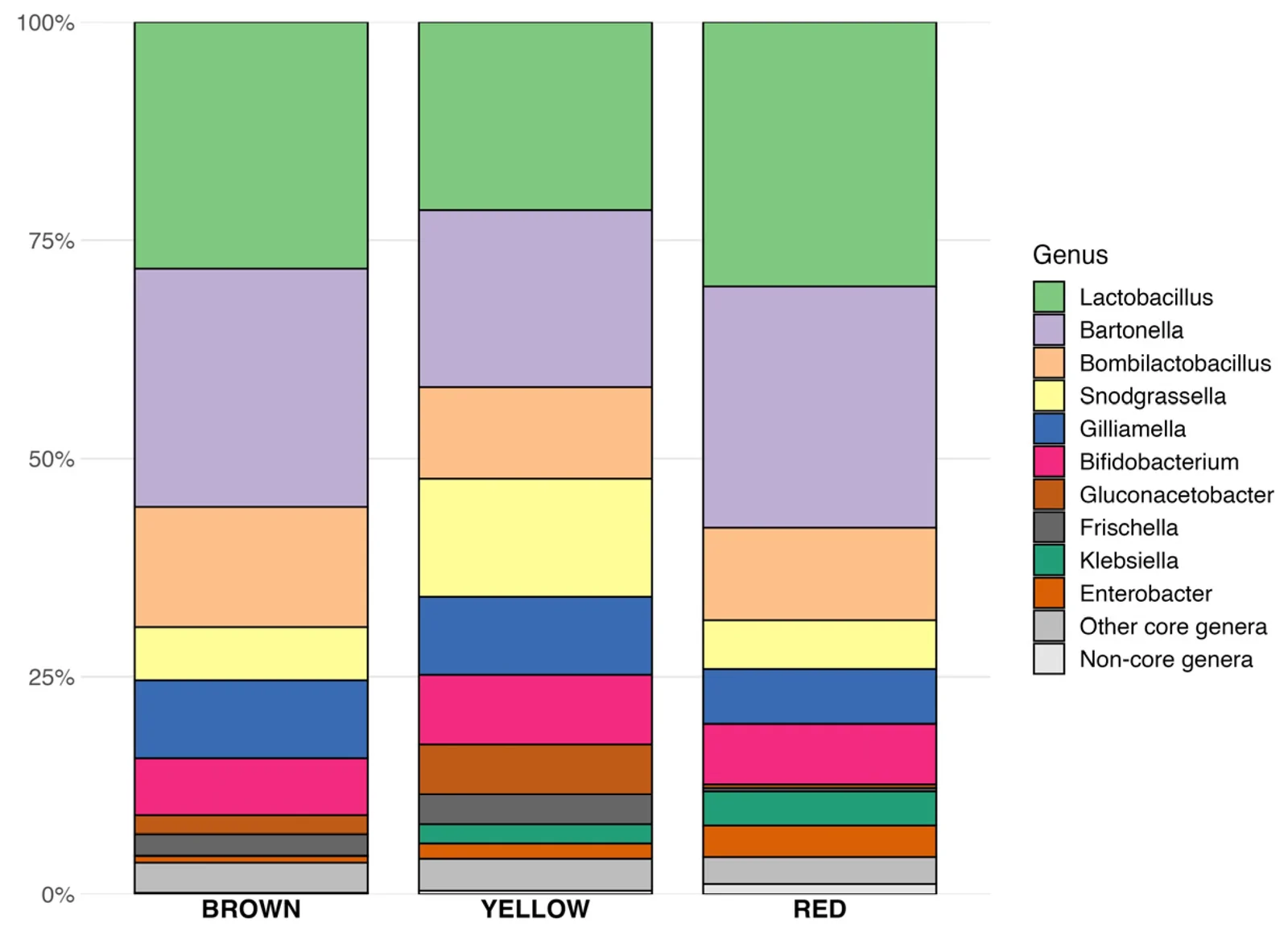
Stacked bar charts showing the mean relative abundance (%) of bacterial genera across the three PFAS exposure-risk areas (Red = high; Yellow = intermediate; Brown = low). The study-specific core microbiome was defined as genera detected in ≥80% of biologically independent apiaries and showing a mean relative abundance ≥0.01%. Core genera reaching ≥1% mean relative abundance in at least one exposure-risk area are shown individually; lower-abundance core genera are grouped as “Other core genera”, while genera not meeting the core microbiome criteria are grouped as “Non-core genera”.

**Figure 3:**
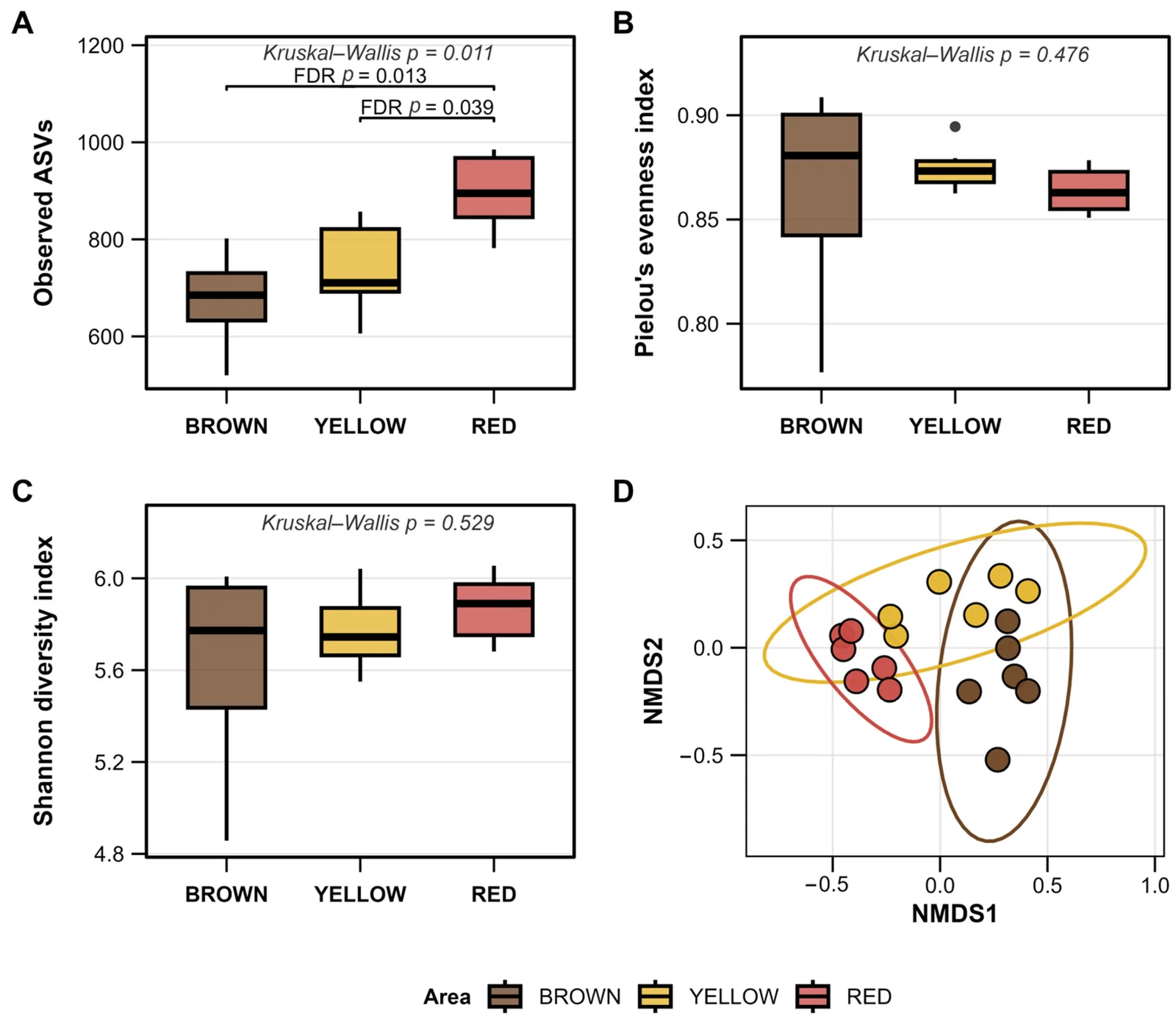
Boxplots of the number of identified ASVs (**A**) and alpha-diversity (**B**,**C**), i.e., Pielou’s and Shannon’s indices, in the pooled honey bees from the three different PFAS exposure-risk areas (Brown, Yellow, Red). Extreme values are indicated by dots. (**D**) Non-metric multidimensional scaling (NMDS) plot of beta-diversity. Each point represents the microbial community of a sample. Samples with a similar microbiota composition tend to cluster in the same area of the plot, while points far apart from each other represent samples with dissimilar microbiota. Kinds of samples are identified by the same color used in the boxplots.

**Figure 4:**
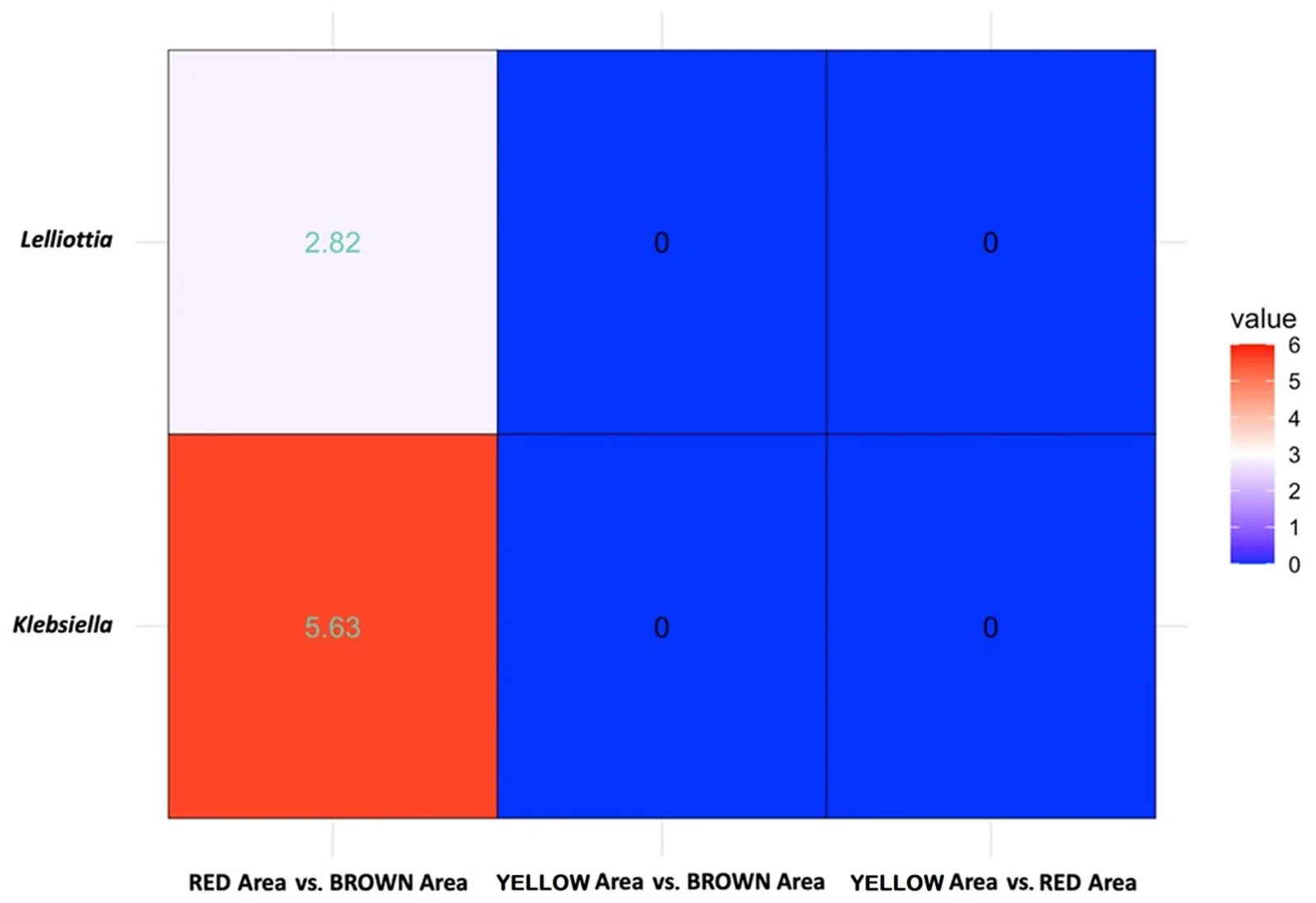
Heatmap of ANCOM-BC pairwise analysis showing the differential *Klebsiella* spp. and *Lelliottia* spp. taxa abundances in the three distinct PFAS exposure-risk areas: high (Red), intermediate (Yellow), and low (Brown). Columns denote the specific comparisons, and the cell colors represent statistically significant changes in bacterial-taxa absolute abundances. The text within each cell represents the log fold-change (LFC) value. Taxa with a prevalence <10% were excluded from the analysis, and only differences with Holm-adjusted *p* < 0.05 are shown. Robustness of significant results was assessed using the ANCOM-BC pseudo-count sensitivity analysis.

**Figure 5:**
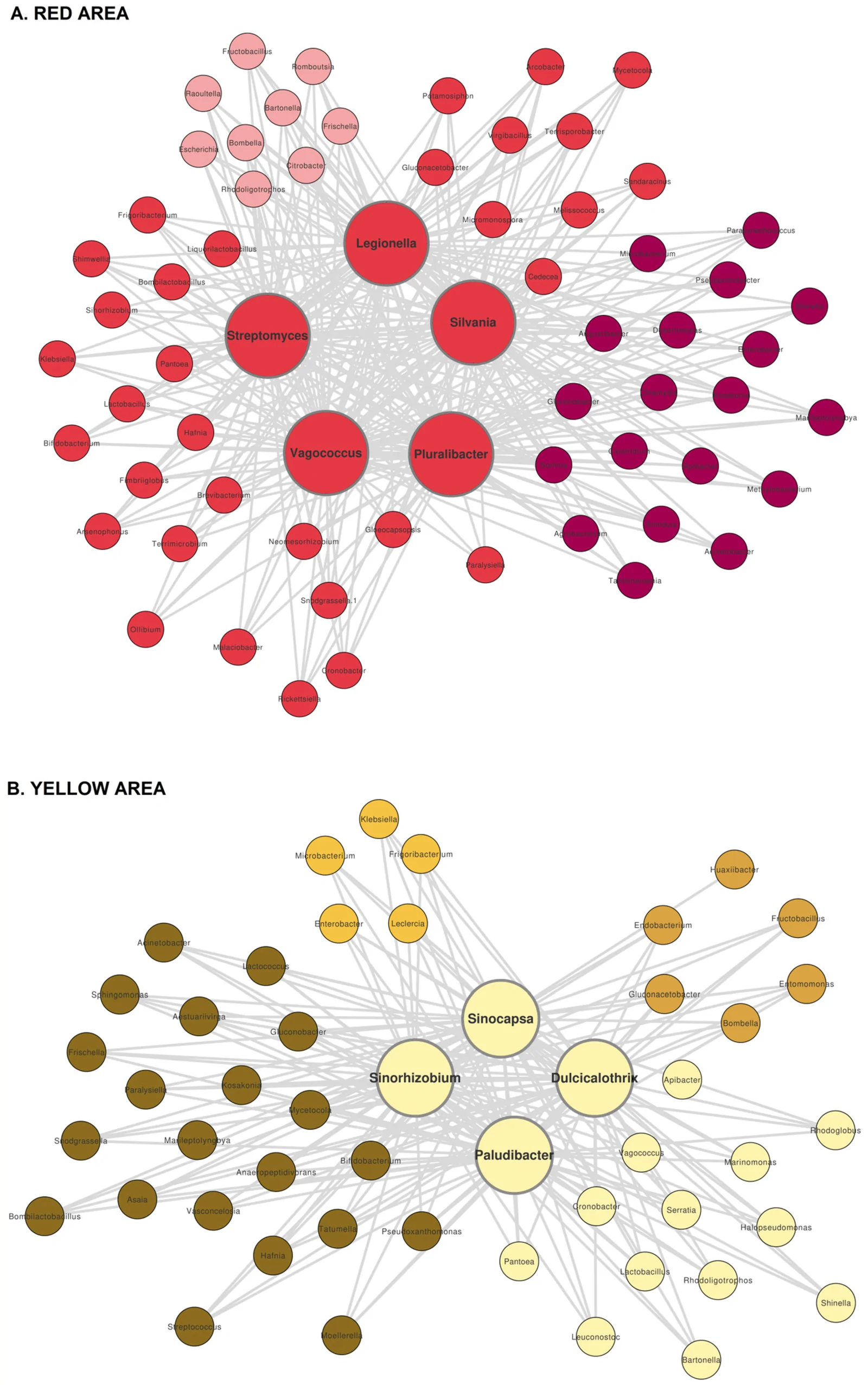

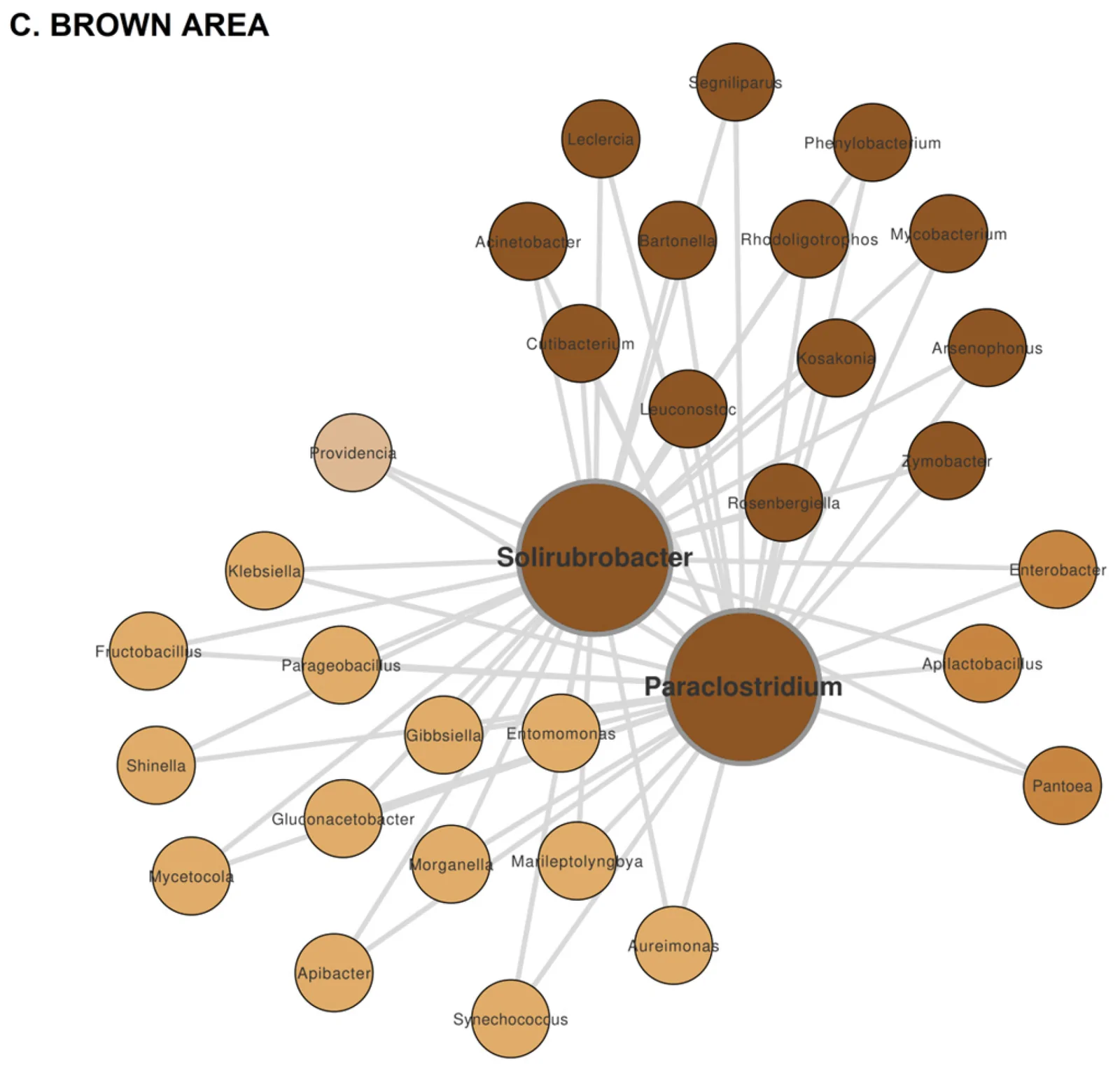
Exploratory hub-centered bacterial-association subnetworks of the honey bee gut microbiota across the three PFAS exposure-risk areas. Genus-level subnetworks are shown for the Red (**A**), Yellow (**B**), and Brown (**C**) areas. Networks were inferred separately for each area using Spearman’s correlations. Nodes represent bacterial genera, whereas grey edges represent retained pairwise associations between genera. Node size reflects eigenvector centrality, with larger black-bordered nodes identifying the principal hub taxa and the genera directly associated with them. Given the limited biological replication (two independent apiaries per exposure-risk area), these networks are presented as exploratory visualizations and the observed network properties and central taxa should be interpreted descriptively.

**Figure 6:**
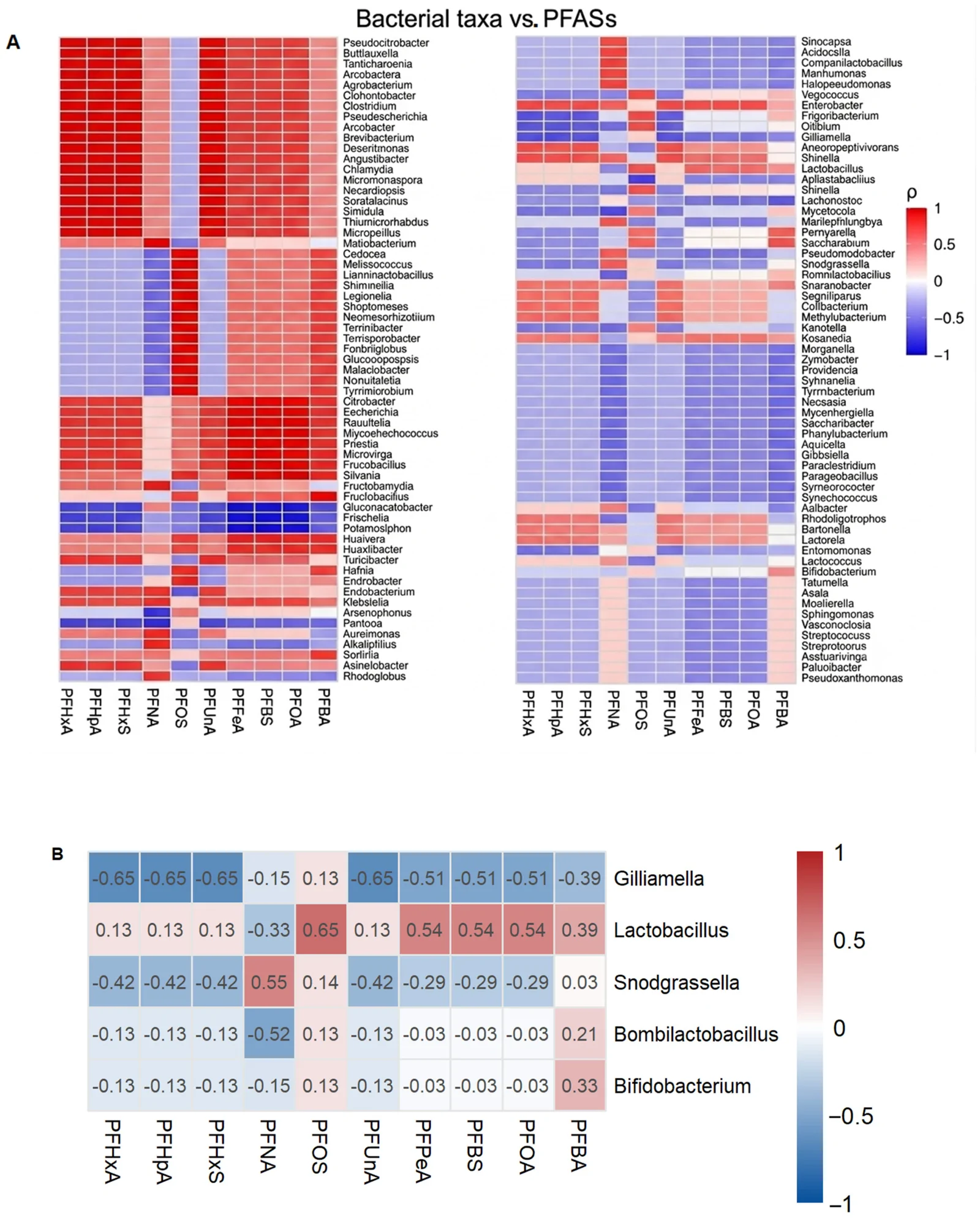
Spearman’s correlation heatmap showing the relationships between individual PFAS concentrations and the relative abundance of all detected bacterial genera (**A**) and bacterial genera belonging to the honey bee core gut microbiota (**B**). Positive correlations are shown in red and negative correlations in blue.

**Table 1:** Target PFAS concentrations (determined by using LC–MS/MS protocols) in honey bees sampled in fifteen apiaries located in three areas with high, intermediate, and low risk of PFAS exposure (Red, Yellow, and Brown areas, respectively). n.d.: not detected.

PFAS Exposure-Risk Area	Apiary Identification	PFAS (µg kg^−1^)													
		PFBA	PFPeA	PFBS	PFHxA	PFHpA	PFHxS	PFOA	PFNA	PFDA	PFOS	PFUnA	PFDoA	GenX	C_6_O_4_
High	H−VI−1	1.924	1.025	0.963	0.87	0.302	0.87	3.03	0.155	n.d.	n.d.	0.06	n.d.	n.d.	n.d.
	H−VI−2	0.196	0.11	n.d.	n.d.	n.d.	n.d.	n.d.	n.d.	n.d.	n.d.	n.d.	n.d.	n.d.	n.d.
	H−VI−3	0.104	n.d.	n.d.	n.d.	n.d.	n.d.	n.d.	n.d.	n.d.	n.d.	n.d.	n.d.	n.d.	n.d.
	H−VI−4	0.105	n.d.	n.d.	n.d.	n.d.	n.d.	n.d.	0.055	n.d.	n.d.	n.d.	n.d.	n.d.	n.d.
	H−VI−5	0.176	n.d.	n.d.	n.d.	n.d.	n.d.	0.076	0.083	n.d.	n.d.	n.d.	n.d.	n.d.	n.d.
	H−VI−6	0.052	n.d.	n.d.	n.d.	n.d.	n.d.	n.d.	n.d.	n.d.	n.d.	n.d.	n.d.	n.d.	n.d.
	H−VI−7	2.69	0.48	0.078	n.d.	n.d.	n.d.	0.893	n.d.	n.d.	0.051	n.d.	n.d.	n.d.	n.d.
Intermediate	I−VI−8	n.d.	n.d.	n.d.	n.d.	n.d.	n.d.	n.d.	n.d.	n.d.	n.d.	n.d.	n.d.	n.d.	n.d.
	I−PD−1	n.d.	n.d.	n.d.	n.d.	n.d.	n.d.	n.d.	n.d.	n.d.	n.d.	n.d.	n.d.	n.d.	n.d.
	I−PD−2	n.d.	n.d.	n.d.	n.d.	n.d.	n.d.	n.d.	0.163	n.d.	n.d.	n.d.	n.d.	n.d.	n.d.
	I−PD−3	0.09	n.d.	n.d.	n.d.	n.d.	n.d.	n.d.	0.122	n.d.	n.d.	n.d.	n.d.	n.d.	n.d.
Low	L−VR−1	n.d.	n.d.	n.d.	n.d.	n.d.	n.d.	n.d.	n.d.	n.d.	n.d.	n.d.	n.d.	n.d.	n.d.
	L−PD−4	n.d.	n.d.	n.d.	n.d.	n.d.	n.d.	n.d.	0.087	n.d.	n.d.	n.d.	n.d.	n.d.	n.d.
	L−VI−9	n.d.	n.d.	n.d.	n.d.	n.d.	n.d.	n.d.	n.d.	n.d.	n.d.	n.d.	n.d.	n.d.	n.d.
	L−VI−10	n.d.	n.d.	n.d.	n.d.	n.d.	n.d.	n.d.	n.d.	n.d.	n.d.	n.d.	n.d.	n.d.	n.d.

**Table 2:** Pesticide residues, (determined by using GC–MS/MS and LC–MS/MS *in-house* validated protocols) in honey bees sampled in fifteen apiaries located in three areas with high, intermediate, and low risk of PFAS exposure (Red, Yellow, and Brown areas, respectively). n.d.: not detected.

PFAS Exposure-Risk Area	Apiary Identification	Pesticide Residues Detected	Concentration (µg/bee)
High	H-VI-1	Cyazofamid	0.0037
		Zoxamide	0.0016
	H-VI-2	Cyazofamid	0.0012
		Zoxamide	0.0010
	H-VI-3	n.d.	
	H-VI-4	n.d.	
	H-VI-5	Amitraz metabolite_N-(2,4-Dimethylphenyl)-N′-methylformamidine	0.0018
	H-VI-6	Chlorfenvinphos	0.0012
	H-VI-7	Chlorfenvinphos	0.0244
Intermediate	I-VI-8	n.d.	
	I-PD-1	n.d.	
	I-PD-2	n.d.	
	I-PD-3	n.d.	
Low	L-VR-1	Propamocarb	0.0015
		Trifloxystrobin	0.0020
	L-PD-4	n.d.	
	L-VI-9	n.d.	
	L-VI-10	n.d.	

**Table 3:** Results (positive and negative detection) of honey bee pathogens investigated, by using *in-house* biomolecular protocols, in the fifteen apiaries located in three areas with different risks of PFAS exposure (high, intermediate, and low; Red, Yellow, and Brown areas, respectively).

PFAS Exposure-Risk Area	Apiary Identification	*Vairimorpha* spp.	*V.* *apis*	*V.* *ceranae*	*C.* *mellificae*	*L.* *passim*	ABPV	CBPV	DWV	BQCV	SBV	IAPV	KBV
High	H−VI−1	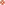	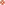	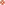	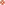	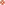	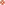	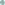	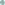	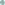	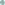	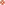	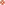
	H−VI−2	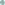	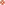	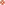	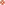	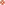	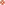	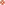	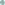	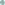	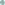	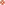	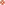
	H−VI−3	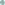	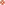	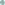	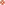	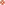	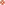	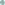	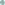	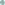	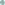	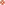	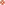
	H−VI−4	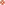	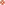	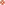	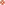	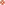	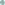	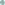	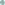	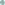	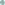	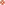	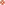
	H−VI−5	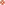	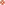	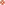	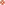	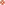	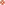	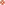	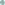	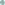	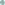	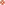	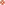
	H−VI−6	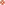	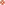	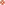	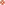	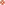	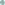	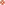	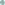	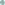	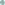	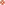	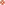
	H−VI−7	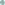	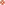	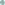	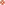	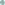	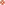	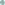	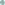	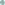	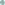	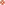	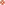
Intermediate	I−VI−8	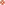	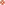	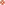	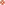	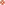	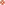	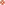	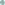	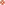	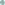	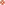	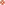
	I−PD−1	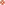	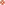	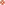	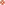	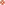	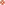	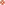	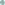	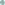	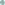	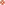	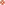
	I−PD−2	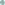	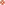	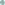	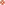	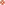	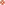	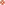	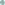	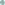	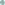	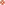	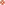
	I−PD−3	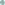	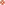	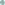	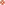	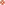	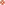	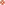	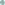	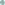	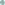	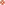	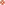
Low	L−VR−1	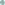	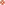	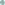	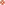	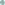	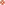	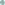	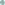	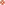	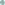	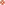	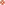
	L−PD−4	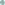	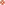	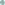	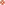	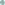	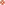	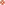	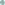	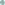	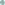	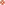	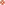
	L−VI−9	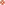	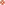	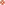	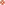	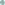	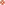	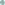	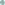	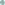	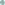	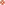	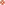
	L−VI−10	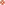	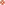	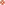	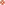	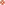	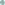	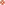	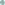	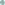	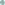	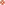	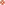
													
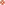	Negative	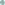	Positive										

**Table 4:** Network-analysis properties (edge density, percentage of positive associations, average dissimilarity, average path length, clustering coefficient, modularity, and natural connectivity) calculated using the netAnalyze in the three PFAS exposure-risk areas (high: Red; intermediate: Yellow; low: Brown).

	Red Area	Yellow Area	Brown Area
**Edge density**	0.620	0.569	0.566
**Positive edge (%)**	63.18	59.70	58.74
**Average dissimilarity**	0.739	0.761	0.765
**Average path length**	0.941	0.958	0.951
**Clustering coefficient**	0.729	0.750	0.708
**Modularity**	0.093	0.161	0.105
**Natural connectivity**	0.266	0.235	0.216

## Data Availability

The DNA raw data generated in this study have been deposited in the Sequence Read Archive (SRA) database (https://www.ncbi.nlm.nih.gov/sra) on 14 August 2026, under the accession number PRJNA1512439.

## Supplementary Materials

The following supporting information can be downloaded at: https://www.mdpi.com/article/10.3390/insects17101027/s1, Figure S1: Presence–absence co-occurrence heatmaps between PFASs and (A) pesticides, (B) pathogens, and (C) bacterial genera across the six sampled apiaries; Table S1: Instrumental mass-spectrometry settings for target compounds; Table S2: Analytical-method validation and quality controls; Table S3: Pesticides and metabolites analyzed by LC–MS/MS and GC–MS/MS techniques (*n* = 292); Table S4: Pesticide residues detected in honey bees collected from the investigated apiaries, corresponding residue concentrations (µg/bee), and acute oral LD_50_ values for *Apis mellifera*; Table S5: Core-/non-core microbiome classification; Table S6: Summary statistics of the qualitative PFAS–feature co-occurrence analysis; Table S7: Spearman correlation coefficients (rho) between individual PFAS concentrations and the relative abundance of all detected bacterial genera; Table S8: Strongest Spearman correlations between PFAS concentrations and bacterial genera.

## Abbreviations

The following abbreviations are used in this manuscript:

DWV	Deformed-Wing Virus
OECD	Organization for Economic Co-operation and Development
PFAS	per- and polyfluoroalkyl substance
PFOA	perfluorooctanoic acid
PFOS	perfluorooctane sulfonic acid
ARPAV	Regional Agency for Environmental Prevention and Protection of Veneto
PFNA	perfluorononanoic acid
PFHxS	perfluorohexane sulfonic acid
PFBA	perfluorobutanoic acid
PFPeA	perfluoropentanoic acid
PFHxA	perfluorohexanoic acid
PFHpA	perfluoroheptanoic acid
PFDA	perfluorodecanoic acid
PFUnA	perfluoroundecanoic acid
PFDoA	perfluorododecanoic acid
PFBS	perfluorobutane sulfonate
GenX	2,3,3,3-tetrafluoro-2-(heptafluoropropoxy)propanoic acid
MPFBA	perfluoro-n-[^13^C_4_]butanoic acid
M5PFPeA	perfluoro-n-[^13^C_5_]pentanoic acid
M5PFHxA	perfluoro-n-[1,2,3,4,6-^13^C_5_]hexanoic acid
M4PFHpA	perfluoro-n-[1,2,3,4-^13^C_4_]heptanoic acid
M8PFOA	perfluoro-n-[^13^C_8_]octanoic acid
M9PFNA	perfluoro-n-[^13^C_9_]nonanoic acid
M6PFDA	perfluoro-n-[1,2,3,4,5,6-^13^C_6_]decanoic acid
M7PFUdA	perfluoro-n-[1,2,3,4,5,6,7-^13^C_7_]undecanoic acid
M2PFDoA	perfluoro-n-[1,2-^13^C_2_]dodecanoic acid
M3PFBS	perfluoro-1-[1,3,4-^13^C_3_]butansolfonate
M3PFHxS	perfluoro-1-[1,3-^13^C_3_]esanosolfonate
M8PFOS	perfluoro-1-[^13^C_8_]octansolfonate
ACN	acetonitrile
MeOH	methanol
QuEChERS	Quick, Easy, Cheap, Effective, Rugged, and Safe
SPE	Solid Phase Extraction
PSA	Primary Secondary Amine
LC–MS/MS	Liquid Chromatography–Tandem Mass Spectrometry
MP B	Mobile Phase B
GC–MS/MS	Gas Chromatography–Tandem Mass Spectrometry
PCR	Polymerase Chain Reaction
ABPV	Acute Bee-Paralysis Virus
BQCV	Black-Queen Cell Virus
CBPV	Chronic Bee-Paralysis Virus
SBV	Sacbrood virus
KBV	Kashmir Bee virus
IAPV	Israeli Acute-Paralysis Virus
16S rRNA	16S ribosomal RNA
ASV	Amplicon Sequence Variant
SRA	Sequence Read Archive
FDR	False Discovery Rate
PERMANOVA	Permutational Analysis of Variance
LFC	Log Fold Changes
PPP	Plant Protection Product
6:2 Cl-PFESA	6:2 chlorinated polyfluoroalkyl ether sulfonic acid
