# Supplementary material for: Honey Bees as Bioindicators of Environmental PFASs Contamination and Potential Effects on Gut-Microbial Communities
Source: Insects. 2026 Oct 6;17(10):1027. doi: 10.3390/insects17101027 (PMC13704952; doi:10.3390/insects17101027)

Supplementary materials

Chromatographic condition for LC-MSMS Analysis

LC-MS/MS analysis was performed on a Shimadzu HPLC system (Kyoto, Japan) coupled to an API 6500 AB SCIEX (Framingham, MA, USA) Triple Quadrupole. The sample (2 μL) was injected into a Waters Acquity UPLC BEH Shield RP 18 (2.1×100 mm), 1.7 μm column (Waters s.r.l., Sesto San Giovanni, Milan, Italy).
The eluents consisted of water with 10 mM ammonium acetate (mobile phase A, MP A) and MeOH/ACN (80/20) with 10 mM ammonium acetate (MP B). The gradient applied was: 0–0.1 min of 5% MP B, then MP B was increased linearly to reach 95% MP B at 8 min and kept for two minutes. Finally, the concentration of MP B was decreased to 5% between 10 and 10.5 min, and the column was equilibrated to initial conditions until 14 min. The flow rate was 0.3 mL/minute, and the column oven temperature was set at 30 °C.
The MS/MS instrument was operated in negative ESI mode, the gas temperature and the ion spray voltage were kept at 350 °C and −2500 V, respectively. Ions were monitored using a multiple reaction monitoring (MRM) mode. Instrumental MS/MS settings and transition ions for each target analyte are reported in Table S1.

Tables

Table S1. Instrumental mass spectrometry settings for target compounds

Compound	Precursor ion (m/z)	Product ion 1 (m/z)	Collision energy (eV)	Product ion 2 (m/z)	Collision energy (eV)	Retention time (minute)	Internal standard
PFBA	213.0	169.1	-14			5.1	MPFBA
PFPeA	217.0	172	-14			8.8	M5PFPeA
PFHxA	313.0	269.0	-13	119.1	-30	10.0	M5PFHxA
PFHpA	363.0	319.4	-15	169.2	-20	10.8	M4PFHpA
PFOA	413.3	369.0	-15	169.0	-20	11.3	M8PFOA
PFNA	463.0	418.9	-13	168.9	-26	11.8	M9PFNA
PFDeA	513.3	469.2	-15	218.9	-22	12.2	M6PFDA
PFUnA	563.0	519.0	-16	319.0	-28	12.5	M7PFUdA
PFDoA	613.0	569.0	-20	318.9	-28	12.8	M2PFDoA
PFBS	299.0	80	-70	98.8	-40	9.5	M3PFBS
PFHxS	399.1	79.9	-85	98.9	-80	11.0	M3PFHxS
PFOS	499.0	80.0	-106	99.0	-80	11.9	M8PFOS
GenX	328.5	284.9	-8	168.9	-17	10.3	M5PFHxA
C6O4	338.9	179.0	-6	112.9	-13	10.6	M5PFHxA
MPFBA	217.0	172.0	-14			5.1	
M5PFPeA	268.0	223.0	-12			8.8	
M5PFHxA	318.0	273.0	-13			10.0	
M4PFHpA	367.0	322.0	-15			10.8	
M8PFOA	421.0	376.0	-14			11.3	
M9PFNA	472.0	427.0	-15			11.8	
M6PFDA	519.0	474.0	-15			12.2	
M7PFUdA	570.0	525.0	-16			12.5	
M2PFDoA	614.9	569.9	-16			12.8	
M3PFBS	302.0	322.0	-15			9.5	
M3PFHxS	402.0	99.0	-70			11.0	
M8PFOS	507.0	99.0	-80			11.9	

Table S2. Analytical method validation and quality controls.

Analytical method was fully validated and the following parameters were evaluated: specificity, linearity, recovery, repeatability, limit of quantification (LOQ). Specificity was assessed in blank matrix by verifying the absence of signal higher than 30% of the LOQ level. Linearity was studied by means of calibration curves constructed with seven levels (including zero level) within the range of 0.125-7.5 μg L^{-1} (corresponding to 0.025 – 1.5 μg kg^{-1} in the sample). Calibration curves were built by plotting the instrument signal versus the analyte concentration. Linear regression analysis was carried out, and the linear calibration model was verified by correlation coefficients (Pearson's ρ) better than 0.992 and by the Mandel test. Deviation from back-calculated concentration from true concentration was less or equal to 20%. The matrix effect was studied by comparing the slope obtained in solvent calibrations with the corresponding matrix-matched calibration at the same concentrations, verifying the signal suppression or enhancement (not more than 20%). Trueness and precision were estimated by analyzing seven replicates at three concentration levels (see table below). LOQ was estimated as the lowest spike level meeting the method criteria for recovery (70–120%) and precision (CV% ≤ 20%). Two negative quality control (QC) samples, one that included all analytical steps in the absence of the original matrix (QC-), and a second consisting of a fully processed blank matrix sample (QCM-), and two positive QC samples, i.e., two blank matrix matched samples, spiked at 0.05 μg kg^{-1} for PFBA and 0.10 μg kg^{-1} for GenX were processed and analyzed in each analytical series to verify absence of contamination and method performance. Whenever the concentration of the analysed sample was out the linearity range of the method, samples were properly diluted with the same matrix extract used to prepare negative quality control (QCM-).

**Compound**	**Nominal Concentration (ng kg^{-1})**	**Recovery (%)**	**Repeatability (%)**
PFBA	50*	103.6	7.6
	100	103.1	16.9
	5000	99.7	6.9
PFPeA	100*	94.6	11.3
	5000	103.2	5.0
PFBS	50*	90.5	13.3
	100	96.6	13.7
	5000	112.8	7.8
PFHxA	50*	101.8	6.9
	100	107.6	10.7
	5000	103.4	5.5
PFHpA	50*	112.9	11.4

	100	111.0	9.5
	5000	105.2	5.3
PFHxS	50*	108.1	6.4
	100	103.3	11.5
	5000	111.3	8.2
PFOA	50*	109.5	7.1
	100	106.0	10.7
	5000	105.9	6.7
PFNA	50*	97.7	15.0
	100	107.4	10.8
	5000	108.1	6.9
PFUnA	50*	100.0	9.4
	100	107.8	13.4
	5000	104.0	9.0
PFDeA	50*	96.4	10.5
	100	101.0	13.4
	5000	107.0	7.3
PFOS	50*	107.8	12.9
	100	100.8	11.2
	5000	111.4	7.0
PFDoA	50*	110.0	6.2
	100	104.4	11.0
	5000	107.6	7.7
GenX	100*	108.2	18.5
	5000	113.4	19.7
	20000	96.6	7.3
C6O4	5000*	105.5	14.6
	20000	96.4	8.8

*Limit of Quantification

Table S3. Pesticides and metabolites analyzed by LC-MS/MS and GC-MS/MS techniques (*n* = 292)

**List of pesticides and metabolites analysed**
2-Phenylphenol; 4,4'-dichlorobenzophenone; Acephate; Acetamiprid; Acetochlor; Acrinathrin; Alachlor; Aldicarb; Aldicarb sulfoxide; Aldicarb-sulfone; Aldrin; Ametryn; Aminocarb; Amitraz; Anthraquinone; Atrazine; Avermectin; Azinphos-ethyl; Azinphos-methyl; Azoxystrobin; Benalaxyl; Bendiocarb; Benzoximate; BHC, alfa; BHC, beta; BHC, delta; BHC, gamma- Lindano; Bifenazate; Bifenthrin; Bitertanol; Boscalid; Bromfenvinphos; Bromfenvinphos-methyl; Bromophos; Bromophos-ethyl; Bromopropylate; Bromucanozole; Bupirimate; Buprofezin; Butafenacil; Butocarboxim; Butoxycarboxim; Carbaryl; Carbendazim; Carbetamide; Carbofuran; Carbofuran-3-hydroxy; Carboxin; Carfentrazone-ethyl; Chlorantraniliprole; Chlorbenzylate; Chlorfenson; Chlorfenvinphos; Chlorfluazuron; Chloroneb; Chloropyriphos-methyl; Chlorotoluron; Chloroxuron; Chlorpropham; Chlorpyrifos; Chlorthal-dimethyl; Chlorthiophos; Chlozolinate; Clethodim; Clomazone; Clothianidin; Coumaphos; Cyazofamid; Cycluron; Cyfluthrin; Cyhalothrin, lambda; Cypermethrin; Cyproconazole; Cyprodinil; Deltamethrin; Desmedipham; Di-allate; Diazinone; Diclobutrazol; Dicrotophos; Diethofencarb; Difenoconazole; Diflubenzuron; Dimethachlor; Dimethoate; Dimethomorph; Dimoxystrobin; Dinotefuran; Dioxacarb; Diphenamid; Disulfoton; Diuron; Edifenphos; Emamectin; Endosulfan sulfate; EPN; Epoxiconazole; Eprinomectin; Ethion; Ethirimol; Ethofumesate; Ethylan; Etofenprox; Etoxazole; Etridiazole; Fenamidone; Fenamiphos; Fenarimol; Fenbuconazole; Fenchlorphos; Fenitrothion; Fenobucarb; Fenoxycarb; Fenpropathrin; Fenpyroximate; Fenson; Fenthion; Fenuron; Fenvalerate; Fipronil; Fipronil-sulfone; Fluazifop-butyl; Fluazinam; Flubendiamide; Flucythrinate; Fludioxonil; Flufenacet; Flufenoxuron; Fluometuron; Fluoxastrobin; Fluquinconazole; Flusilazole; Flusilazole; Flutolanil; Flutriafol; Fluvalinate; Fonofos; Forchlorfenuron; Formetanate hydrochloride; Fuberidazole; Furalaxyl; Furathiocarb; Halofenozide; Hexaconazole; Hexaflumuron; Hexazinone; Hexythiazox; Hydramethylnon; Imazalil; Imidacloprid; Indoxacarb; Iodfenphos; Ipconazole; Iprodione; Iprovalicarb; Isazophos; Isocarbofos; Isodrin; Isoprocarb; Isoproturon; Ivermectine; Kresoxim-methyl; Lenacil; Leptophos; Linuron; Lufenuron; Malathion; Mandipropamid; Mefenacet; Mepanipyrim; Mepronil; Metalaxyl; 2,4-dimethylaniline; N-(2,4-Dimethylphenyl)formamide; N-(2,4-Dimethylphenyl)-N'-methylformamidine; Metconazole; Methabenzthiazuron; Methacrifos; Methamidophos; Methiocarb; Methomyl; Methoprotryne; Methoxychlor; Methoxyfenozide; Methyl parathion; Metobromuron; Metolachlor; Metribuzin; Mevinphos ; Mexacarbate; MGK 264; Mirex; Monocrotophos; Monolinuron; Myclobutanil; Neburon; Nitenpyram; o,p'-DDD; Omethoate; Oxadiazon; Oxadixyl; Oxamyl; Paclobutrazol; Parathion; Penconazole; Pencycuron; Pentachlorobenzene; Permethrin; Phenmedipham; Phenothrin; Phorate; Phosalone; Phosmet; Picoxystrobin; Piperonyl butoxide; Pirimicarb; Pirimiphos ethyl; Pirimiphos methyl; Pretilachlor; Prochloraz; Procymidone; Profenofos; Promecarb; Prometon; Prometryn; Propachlor; Propamocarb; Propanil; Propargite; Propham; Propiconazole; Propisochlor; Propoxur; Propyzamide; Prothiofos; Pymetrozine; Pyracarbolid; Pyraclofos; Pyraclostrobin; Pyrazophos; Pyridaben; Pyridafenthion; Pyrimethanil; Pyriproxyfen; Quinalphos; Quinoxyfen; Rotenone; Secbumeton; Siduron; Simetryn; Spinetoram; Spinosad; Spirodiclofen; Spiromesifen; Spirotetramat; Spiroxamine; Sulfotep; Sulprofos; Tebuconazole; Tebufenozide; Tebufenpyrad; Tebuthiuron; Teflubenzuron; Tefluthrine; Terbacil; Terbufos; Terbumeton; Terbuthylazine; Terbutryn; Tetrachlorvinphos; Tetraconazole; Tetradifon; Tetramethrin; Thiabendazole; Thiacloprid; Thiamethoxam; Thidiazuron; Thiobencarb; Thiophanate-methyl; Tolclofos-methyl; Transfluthrin; Triadimefon; Triadimenol; Triazophos; Tricyclazole; Trifloxystrobin; Triflumizole; Trillate; Triticonazole; Vamidothion; Vinclozoline; Zoxamide.

Table S4. Pesticide residues detected in honey bees collected from the investigated apiaries, corresponding residue concentrations (μg/bee), and acute oral LD_{50} values for *Apis mellifera*

Active substance	Action	Detected concentration (μg/bee)	Contact acute LD_{50} (μg/bee)*	Oral acute LD_{50} (μg/bee)*
Amitraz	acaricide/insecticide	0.0018	50	n.a.
Chlorfenvinphos	insecticide	0.0012-0.0244	4.1	0.55
Cyazofamid	fungicide	0.0012-0.0037	> 100	>151.7
Propamocarb	fungicide	0.0015	> 100	>84
Trifloxystrobin	fungicide	0.0020	> 200	>200
Zoxamide	fungicide	0.0010-0.0016	> 100	>147

n.a.: data not applicable

* obtained from the Pesticide Properties DataBase (PPDB), University of Hertfordshire, UK.

Figures

Figure S1. Presence–absence co-occurrence heatmaps between PFASs and (**A**) pesticides, (**B**) pathogens and (**C**) bacterial genera across the six sampled apiaries. Cell colour indicates the number of apiaries in which each PFAS–feature pair co-occurred, ranging from 0 (white) to 6 (black). The analysis is descriptive and based on binary presence–absence data.

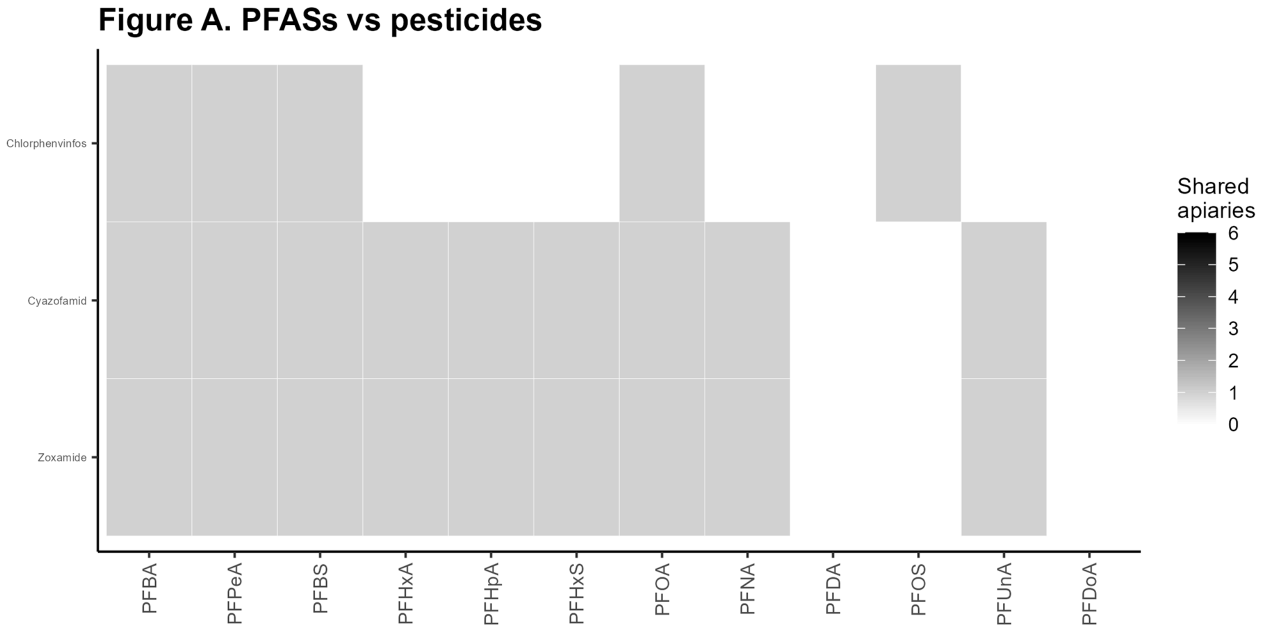

Figure B. PFASs vs pathogens

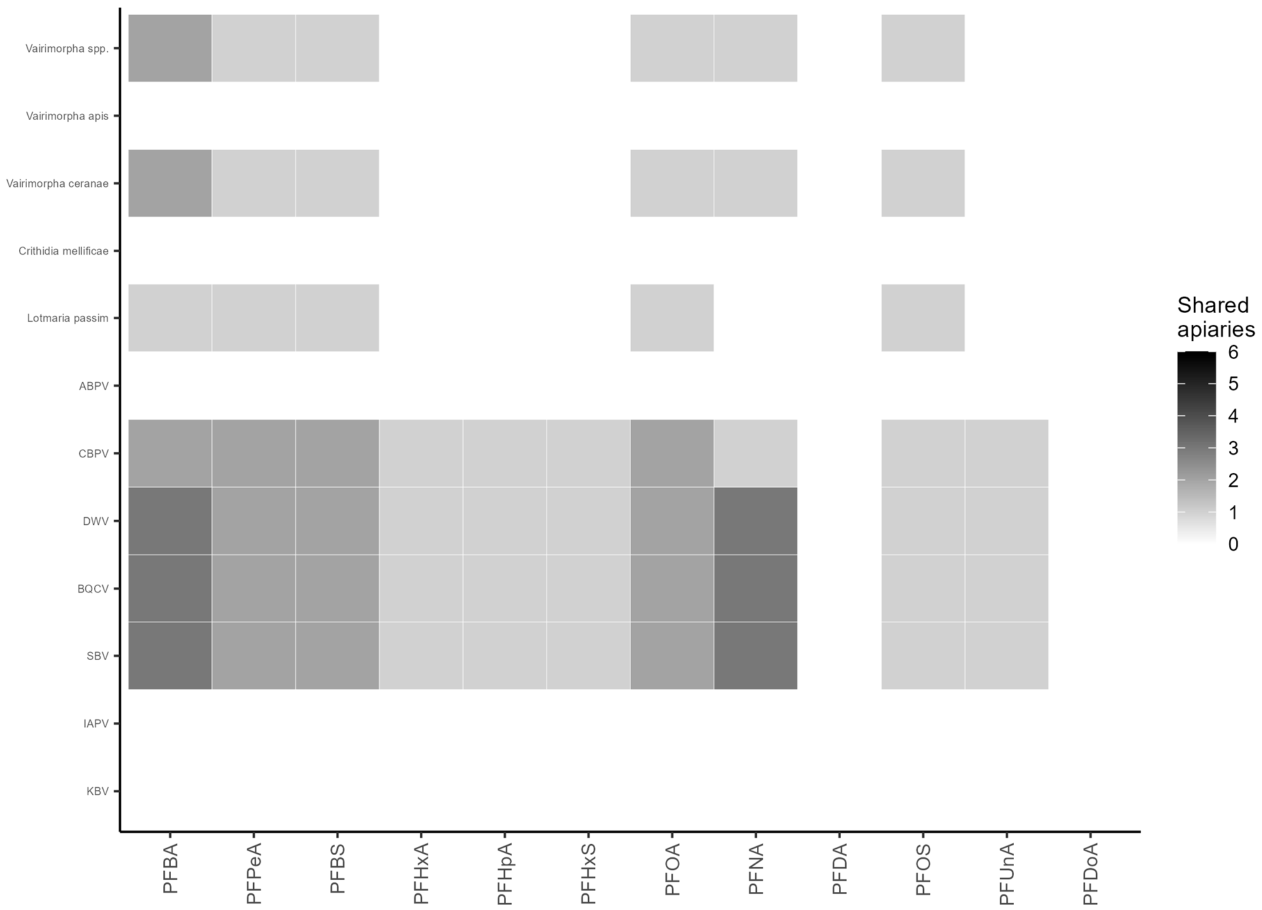

Figure C. PFASs vs all bacterial genera

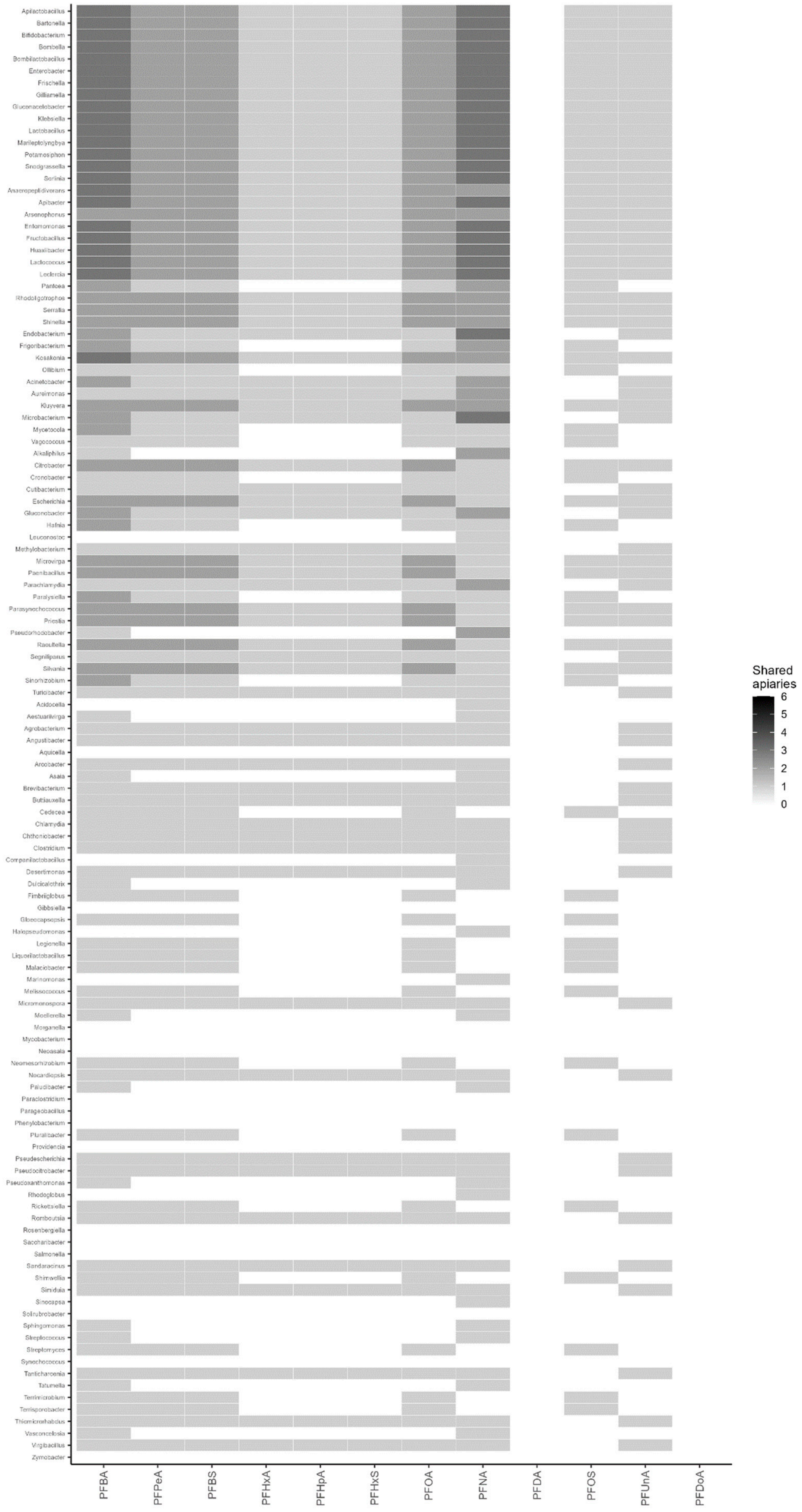

Supplement: Supplementary file 1 [file insects-17-01027-s001.zip › Supplementary materials_analytical chemistry and statistical figures_revised_modified.pdf]
